# Exploiting a novel conformational switch to control innate immunity mediated by complement protein C3a

**DOI:** 10.1038/s41467-017-00414-w

**Published:** 2017-08-24

**Authors:** Rink-Jan Lohman, Johan K. Hamidon, Robert C. Reid, Jessica A. Rowley, Mei-Kwan Yau, Maria A. Halili, Daniel S. Nielsen, Junxian Lim, Kai-Chen Wu, Zhixuan Loh, Anh Do, Jacky Y. Suen, Abishek Iyer, David P. Fairlie

**Affiliations:** 1 Institute for Molecular Bioscience, The University of Queensland, Brisbane, QLD 4072 Australia; 2 Centre for Inflammation and Disease Research, The University of Queensland, Brisbane, QLD 4072 Australia; 3 Australian Research Council Centre of Excellence in Advanced Molecular Imaging, The University of Queensland, Brisbane, QLD 4072 Australia

## Abstract

Complement C3a is an important protein in innate and adaptive immunity, but its specific roles in vivo remain uncertain because C3a degrades rapidly to form the C3a-desArg protein, which does not bind to the C3a receptor and is indistinguishable from C3a using antibodies. Here we develop the most potent, stable and highly selective small molecule modulators of C3a receptor, using a heterocyclic hinge to switch between agonist and antagonist ligand conformations. This enables characterization of C3 areceptor-selective pro- vs. anti-inflammatory actions in human mast cells and macrophages, and in rats. A C3a receptor-selective agonist induces acute rat paw inflammation by first degranulating mast cells before activating macrophages and neutrophils. An orally administered C3a receptor-selective antagonist inhibits mast cell degranulation, thereby blocking recruitment and activation of macrophages and neutrophils, expression of inflammatory mediators and inflammation in a rat paw edema model. These novel tools reveal the mechanism of C3a-induced inflammation and provide new insights to complement-based medicines.

## Abstract (web summary)

Complement C3a is an important protein in innate and adaptive immunity, but its roles in vivo are unclear. Here the authors develop novel chemical agonists and antagonists for the C3a receptor, and show that they modulate mast cell degranulation and inflammation in a rat paw edema model

## Introduction

Protein-protein interactions (PPIs) mediate most physiological processes and involve large interacting protein surfaces that are proving to be extremely difficult to mimic, or interfere with, using small molecules in vivo^1, 2^. Most PPIs are currently thought to be ‘undruggable’ using conventional small drug-like molecules^1–3^. A significant challenge in chemical biology is to rationally downsize a protein to an equipotent small molecule that could be cheaper to manufacture, easier to structurally and functionally modify, non-immunogenic, and likely more stable and more orally active^1, 4^. Complement C3a is a ~ 9 kDa helix bundle inflammatory protein that binds to a ~ 100 kDa G protein-coupled receptor called C3aR expressed on the cell surface. C3a is thought to be important in mediating inflammatory responses to infection and injury^4–6^. C3a concentrations are reportedly elevated during inflammatory diseases^7^, recombinant C3a reportedly induces hypertension and delayed neutrophilia in rats over 24 h^8^, while sustained activation of C3aR vs knockouts support a role in allergies^9^, asthma^10^, arthritis^11^, sepsis^12^, lupus^13^, diabetes^14^, ischemia-reperfusion injury^15^, obesity and metabolic dysfunction^16^. However, the actions of C3a in vivo remain uncertain because C3a is synthesized at the cell surface and very rapidly degraded by extracellular carboxypeptidases, which cleave off the C-terminal residue Arg to form C3a des-Arg^4–6^ that does not bind to C3aR and has a completely different pharmacological profile. Moreover, commercially available antibodies used in many in vivo studies do not discriminate between C3a and C3a-desArg^7, 15^. Thus, most claims for detection of C3a in vivo or for properties of exogenous C3a administered in vivo may be compromised. Synthetic agonists that act through C3aR, but do not degrade rapidly like C3a, could aid the characterization of C3a biology in vivo and may be valuable immunostimulants or antimicrobial agents^17^, while metabolically stable and orally active antagonists may be valuable new anti-inflammatory agents with therapeutic potential^4–6, 18^. No drug-like small molecule agonists or antagonists of C3aR have been found yet with high potency, selectivity, metabolic stability and oral bioavailability for interrogating C3a-mediated functions in vivo^4^.

Recently, we described an approach to rationally downsizing the 77 residue human complement C3a protein to small molecule agonists (MW < 500) equivalent in size to just the last 3–4 amino acid residues of the C-terminus of C3a^4^. These compounds were built from the C-terminal arginine of C3a and displayed the same capacity in vitro as human C3a to induce calcium release and the expression of inflammatory cytokines in human macrophages^4^. While C3a is degraded within minutes in plasma, these small molecule proxies for C3a are stable in plasma and may be useful with some modifications as pharmacological tools to probe C3a properties in vivo. We have also probed how the relative hydrogen-bonding potential of different heterocycles contributes to binding affinity^19^ and used theoretical calculations to predict barriers to rotation, and hence the probable population of different conformers which was then related to functional activity^20^. Here we have significantly extended that work by incorporating different heterocycles to switch from agonist to antagonist conformations, culminating in the most potent small molecule activators and inhibitors known for the C3a receptor in vitro and in vivo. We characterize their solution structures, their activities on human mast cells and macrophages, their target specificity, and their effects on C3a-dependent innate immune responses in a rat model of acute inflammation. This is an important advance in (i) identifying the actions of the C3a protein, which degrades rapidly in biological fluids, (ii) rationally developing small molecule agonists and antagonists of C3aR for use in vivo in physiology and disease, and (iii) identifying the temporal sequence of cellular immune responses to activation of C3aR in rodents. This novel approach to downsizing a protein to conformationally restricted small molecules may lead to complement-based medicines and encourage similar approaches to modulate other protein–protein interactions.

## Results

### Heterocycles switch ligand function on human mast cells

**Fig. 1:**
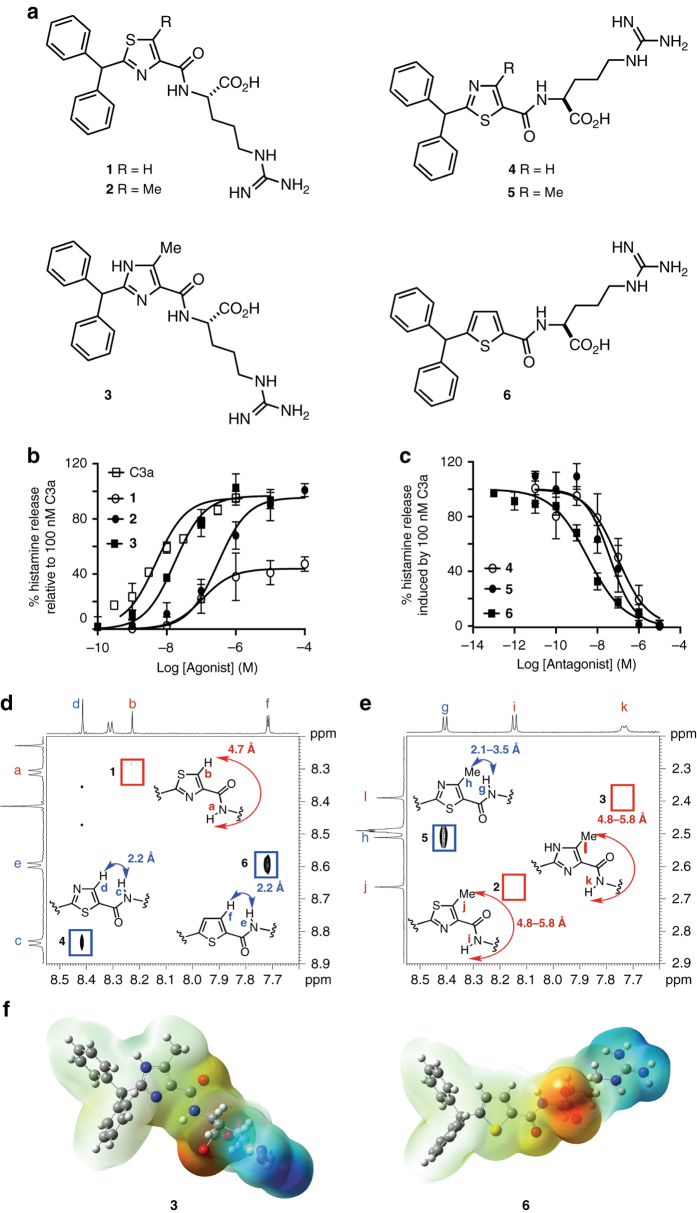
Structures and activities for human C3aR agonists and antagonists. **a** Chemical structures of synthetic compounds **1**–**6**; **b**, **c** Concentration-dependent histamine secretion in human LAD2 mast cells induced by: **b** hC3a (EC_50_ 3 ± 1.5 nM) vs. agonists **1** (EC_50_ 110 ± 40 nM), **2** (EC_50_ 300 ± 100 nM) or **3** (EC_50_ 20 ± 10 nM) alone. **c** hC3a (100 nM) was inhibited by antagonist **4** (IC_50_ 90 ± 50 nM) or **5** (IC_50_ 40 ± 20 nM) or **6** (IC_50_ 3.5 ± 1.5 nM). All the data *n* ≥ 3, *error bars* are ± SEM; **d**, **e** 2D ROESY ^1^H-NMR spectra in DMSO-d_6_ for **1**–**6**. **d** Amide NH…aromatic CH ROE correlations (c,d and e,f labeled protons, *blue boxes*) for antagonists **4** and **6**, but not for agonist **1** (a,b labeled protons expected in *red box*). **e** 2D ROESY ^1^H-NMR spectra in DMSO-d_6_ show amide NH…Me ROE correlations for antagonist **5** (h, g labeled protons, *blue box*) but not for agonists **2** and **3** (i,j or k,l labeled protons expected in *red boxes*). The data indicate a distinct conformational difference between agonists (**1**, **2**, **3**) and antagonists (**4**, **5**, **6**). **f** Energy minimized structures with electrostatic surface potential maps for agonist **3** and antagonist **6** show the respective X-C-C-O dihedral angles and dramatically different orientations of the Arg residue. Ab initio calculations (DFT B3LYP/6-311g(2d,2p)) were performed using Gaussian 09 and imaged with GaussView 5

Human complement protein C3a is known to degranulate mast cells in vitro to release histamine^21^. Here we report (Fig. 1a) a thiazole-containing small molecule **1** (R=H), which is a partial agonist at sub-µM concentrations in inducing histamine release from human LAD2 mast cells (Fig. 1b). Incorporating a 5-methyl substituent (R=Me) gives the thiazole analogue **2**, which shows a full agonist response (Fig. 1b). Replacing the sulfur atom in **2** with an NH, to produce the alternative imidazole heterocycle in compound **3**, increases agonist potency by a further log unit, leading to potency comparable to human C3a itself (Fig. 1b). Interestingly, we find that transposing the positions of nitrogen and sulfur atoms in the central thiazole ring of compounds **1** and **2** to give compounds **4** and **5**, respectively (Fig. 1a) switches the biological function from agonism (Fig. 1b) to antagonism of C3a-induced histamine secretion (Fig. 1c). Thiazoles **4** and **5** were the most potent C3a antagonists known, the methylated analogue **5** being slightly less potent. Removal of the thiazole nitrogen atom in **4** gave the thiophene **6**, which is a 10-fold more potent antagonist of C3aR (Fig. 1c). Compounds **3** and **6** are the most potent small molecule agonists and antagonists reported for the human C3a receptor.

### Heterocycles switch an agonist to an antagonist conformation

To understand the molecular basis for these functional changes, we investigated the solution structures of these six compounds using 2D ROESY NMR spectroscopy. Two separate ROESY experiments are compared here for the three non-methylated heterocyclic ligands **1**, **4** and **6** (Fig 1d) vs. the three methylated heterocyclic ligands **2**, **3** and **5** (Fig. 1e). The three compounds in each of these experiments were dissolved together in the same NMR tube at a concentration of 3 mM to ensure that agonists and antagonists were compared under the same conditions and that relative ROE intensities were consistent. The antagonist compounds **4**, **5** and **6** all showed a strong ROE correlation for the amide-NH…H or CH_3_ of heterocycle, indicating close proximity and a S-C-C-O dihedral angle of ~ 0°. Through-space distances of 2.1–3.5 Å (for different methyl group rotamers) were measured from molecular models. Conversely, agonists **1**, **2** and **3** showed no amide-NH….H or CH_3_-heterocycle cross-peaks (expected in the boxed regions, Fig. 1e, f) consistent with larger through-space distances. Molecular modeling suggests distances between 4.7 and 5.8 Å, consistent with a N–C–C–O dihedral angle of ~ 180°. This is the first direct experimental evidence revealing that heterocycles in C3a ligands dictate structure by adopting one of two discrete conformations in solution, with the X–C–C–O dihedral angle being 0° or 180° in agreement with theoretical models^20^. A consequence of this heterocycle-induced conformational switch is that the arginine is positioned differently in agonists vs. antagonists (Fig. 1f, *blue* surface). We attribute this conformational switching to an attractive S…O interaction between the thiazole/thiophene sulfur and the amide carbonyl oxygen in **4**–**6**, fixing the carbonyl oxygen in such a conformation that evidently facilitates antagonist activity. This kind of attractive S…O interaction has been shown in crystal structures of other thiazoles and thiophenes^20, 22^. On the other hand, in **1**–**3** there is no attractive heterocycle–N…O–amide carbonyl interaction, instead the opposite amide orientation is preferred and this conformation clearly favors agonist activity. These highly original findings demonstrate that key functional activities (Fig. 1b, c) of heterocyclic ligands acting at the human C3a receptor can be switched by changing the heteroatom and, consequently, the ligand conformation (Fig. 1d–f).

### Heterocycles switch ligand function on human macrophages

**Fig. 2:**
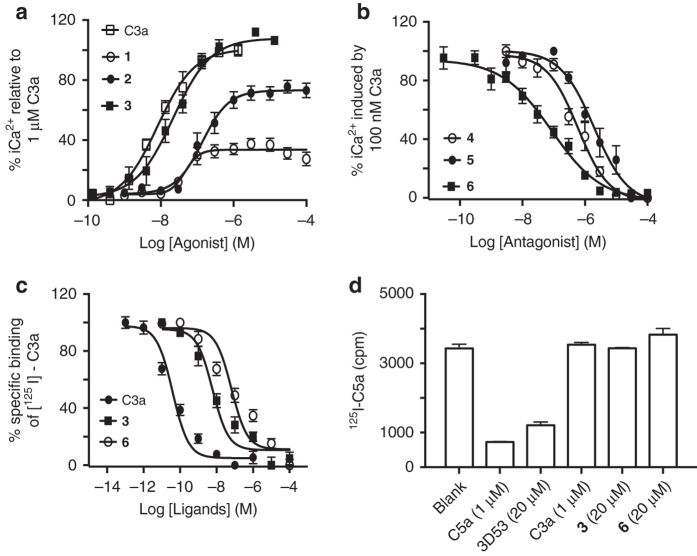
Binding and functional selectivity of C3aR ligands on human macrophages. **a**, **b** Concentration-dependent Ca^2+^ release in human monocyte-derived macrophages (HMDMs) induced by: **a** hC3a (EC_50_ 8 ± 2 nM) and agonists **1** (EC_50_ 50 ± 20 nM), **2** (EC_50_ 140 ± 60 nM) or **3** (EC_50_ 22 ± 8 nM). **b** hC3a (100 nM) induced Ca^2+^ release is inhibited by antagonist **4** (IC_50_ 600 ± 200 nM) or **5** (IC_50_ 2 ± 1.1 μM) or **6** (IC_50_ 85 ± 40 nM); **c** C3aR-binding affinities of hC3a, agonist **3** and antagonist **6** measured by competition with [^125^I]-hC3a (80 pM) on HMDMs. **d** C5aR-binding of hC5a and C5aR selective antagonist (3D53), but no binding of hC3a, agonist **3** or antagonist **6** as measured by competition with [^125^I]-hC5a (25 pM) on HMDMs. All the data *n* ≥ 3, *error bars* are ± SEM

The same functional trends observed for compounds **1**–**6** on histamine secretion in human mast cells (Fig. 1b, c) were also observed for intracellular Ca^2+^ release in human monocyte-derived macrophages, with **1–3** being agonists (Fig. 2a) and **4–6** being antagonists of C3a-induced intracellular Ca^2+^ release (Fig. 2b). The most potent agonist **3** and antagonist **6** were found to compete with ^125^I-labelled C3a (Fig. 2c), but not with ^125^I-labelled C5a (Fig. 2d), for binding to human macrophages, indicating binding and selectivity for C3aR over C5aR. Their reduced affinity relative to human C3a is most likely due to the absence of the high affinity binding N-terminal helix bundle domain of C3a, but this did not affect the functional potency of the small molecules (Fig. 2a, b). This is most likely due to the small molecules binding only at the effector site on C3aR, like the low affinity effector C-terminal tetrapeptide sequence of C3a.

**Fig. 3:**
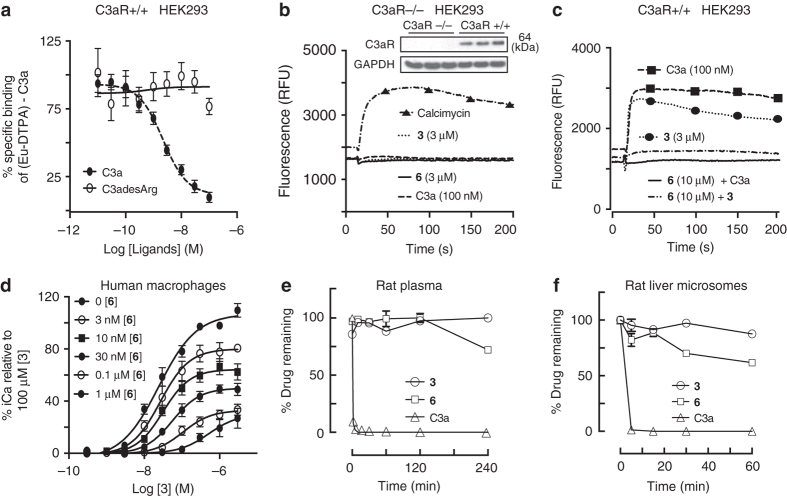
Binding and function of C3aR ligands on HEK293 and HMDM cells. **a** C3aR-binding affinities of hC3a and hC3a-desArg measured by competition with [Eu DTPA]-hC3a (2 nM) on human HEK293 cells transfected with C3aR (C3aR^+/+^); **b** western blot showing no human C3aR expression, and fluorescence plots showing no intracellular Ca^2+^ release induced by C3a, **3** or **6** (vs. positive control: calcimycin) in wild-type human HEK293 cells (C3aR^−/−^); **c** Intracellular Ca^2+^ release induced by C3a or **3** alone or in the presence of **6** (pre-treated 30 min before adding C3a or **3**) in human HEK293 cells transfected with C3aR (C3aR^+/+^); **d** Concentration dependent iCa^2+^ release induced by agonist **3** in the presence of 0–1000 nM antagonist **6** (pre-treated 30 min before adding agonist), relative to 100% response for 100 µM **3** on HMDMs. All the data in **a**–**d**, *n* ≥ 3, *error bars* ± SEM. **e**, **f** Metabolic stability for agonist **3** and antagonist **6** vs hC3a in **e** rat plasma over 4 h and **f** rat liver microsomes over 1 h, illustrating greater stability of **3** and **6** over hC3a

We also checked possible binding of C3a-desArg to C3aR on Human Embryonic Kidney HEK293 cells transfected with human C3aR (C3aR^+/+^) and found no binding (Fig. 3a), indicating that only intact C3a can activate C3aR and that the known removal of the C-terminal arginine of C3a by carboxypeptidases in plasma abrogates C3a activity. Next, we demonstrated the specificity and on-target action of **3** and **6** further in different cell types. Their selectivity for the C3a receptor was indicated by lack of function on wild-type HEK293 cells (C3aR^−/−^), which do not express the C3a receptor^23^ (Fig. 3b) but do endogenously express many other human GPCRs and proteins^24, 25^. C3a, **3** and **6** did not induce Ca^2+^ release, unlike calcimycin, in these C3aR^−/−^ cells (Fig. 3b).

In contrast, both C3a and **3** did induce Ca^2+^ release in human HEK293 cells transfected with C3aR (C3aR^+/+^), consistent with their specificity and on-target action on C3aR (Fig. 3c). This induction in intracellular Ca^2+^ release was prevented by pre-treatment with **6** for 30 min before the addition of either C3a or **3** to C3aR-HEK293 cells (Fig. 3c). Further, the C3a antagonist **6** dose-dependently antagonized intracellular Ca^2+^ release induced by agonist **3** in human macrophages (Fig. 3d). There was a rightward shift in the observed potency of agonist **3** competing with increasing concentrations of **6** in macrophages, with a Schild plot slope of 0.77 ± 0.05 (*r* ^*2*^ = 0.99) (Fig. 3d). The decreasing amplitude of the agonist response with increasing antagonist is consistent with insurmountable antagonism, hinting that **6** may have a longer residence time than **3** on the receptor.

### Plasma and liver microsomal stability

In preparation for evaluating the most potent agonist (compound **3**, designated BR103) and antagonist (compound **6**, designated BR111) in vivo, the in vitro stability of these compounds were examined in rat plasma and in rat liver microsomes. These assays provide a measure of stability towards degradation by proteolytic and cytochrome P450 enzymes, respectively. Compounds **3** and **6** remained intact after exposure to plasma, whereas C3a degraded within minutes in plasma **(**Fig. 3e **)**, a property previously attributed to cleavage by circulating carboxypeptidases^26^. Furthermore, compounds **3** and **6** were much more stable than C3a in liver microsomes, known to be rich in cytochrome P450 enzymes, as reflected by their lower intrinsic clearance rates **(**Fig. 3f **)**. These results suggested that compounds **3** and **6** could potentially be more stable and more bioavailable in vivo than C3a or its peptide analogues, and thus represent important and novel small molecule leads for modulating C3aR biology in vivo.

### Agonist induced acute rat paw edema and immune responses

**Fig. 4:**
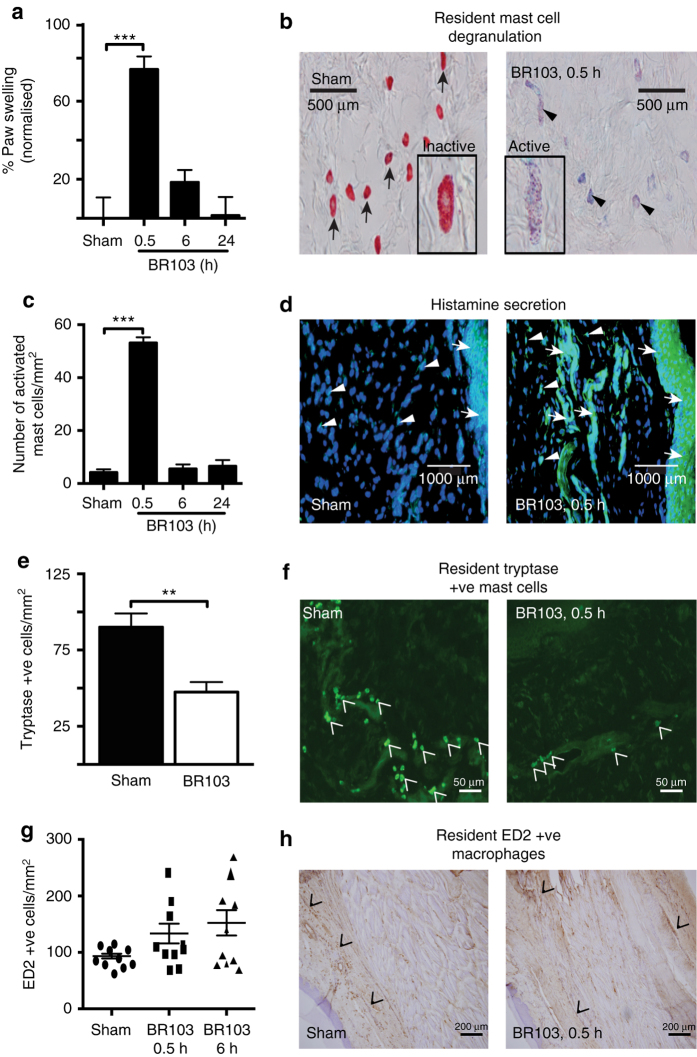
Agonist **3** (BR103) induces paw oedema and mast cell activation in rats. BR103 (350 µg per paw, i.pl.) induces: **a** paw swelling, which peaked at 30 min and returned to near basal levels at 24 h; **b** activation/degranulation mast cells at 30 min stained *purplish blue* by alcian blue (*arrowheads*), whereas inactive mast cells stained red with safranin O (*arrows*), *Scale bar*: 500 μm; **c** Quantification of increased activated mast cells at 30 min, not present at 6 h or 24 h, compared to sham (vehicle) based on staining with alcian blue and safranin O; **d** histamine release at 30 min compared to sham (vehicle), as demonstrated by immunohistochemistry staining of histamine, the blue dye stains for the cell nucleus (*white arrowheads*), while the green dye stains for histamine (*white arrows* – diffused extracellular histamine staining), *Scale bar*: 1000 μm; **e**, **f** tryptase-+ve mast cells are decreased at 30 min compared to sham (vehicle) **e**, as demonstrated in the immunohistochemistry staining of mast cell tryptase **f**, the green dye stains for intact cellular mast cell tryptase in non-degranulated mast cells (*white chevron* – tryptase + ve cells), *Scale bar*: 50 μm; **g**, **h** Quantification **g** and representative images **h** of resident ED2^+^ macrophages (*black chevron*) at 30 min and 6 h compared to sham (vehicle) as demonstrated by immunohistochemistry staining, *Scale bar*: 200 μm. *Error bars* represent mean ± SEM. *P < 0.05; **P < 0.01; ***P < 0.005 (one-way ANOVA, Uncorrected Fisher’s LSD post hoc comparison, a, c, g), student’s t-test e.). 8- to 9-week-old male Wistar rats (*n* = 29) were used for this experiment

C3a-induced inflammatory responses via activation of C3aR have been extensively studied in vitro in human and rodent immune cells^4–6^. However, teasing out the roles of C3a and C3aR in vivo and mechanisms of acute and chronic inflammatory diseases have proven to be more difficult. Studies using recombinant C3a in vivo in rodents are now thought to have been compromised by rapid degradation of C3a due to carboxypeptidase action to form C3a des-Arg^4–6^. C3a des-Arg has a completely different pharmacological profile compared to C3a, and does not bind to C3aR^4–6^, as confirmed above (Fig. 3a). Thus, here we used our more metabolically stable and C3aR-selective small molecule agonist BR103 (compound **3**) to characterize for the first time a C3aR-induced innate immune response in vivo in rats. An acute inflammatory response and edema can be induced in rodents by an intraplantar injection of substances such, as λ-carrageenan into, the rear paws leading to leukocytosis and a hyperemic response, which produces localized swelling^27^. Similarly, we aimed here to investigate if selective activation of C3aR in vivo in rats would induce an acute inflammatory edema and to characterize the innate immune responses over a 24 h time course (Fig. 4).

**Fig. 5:**
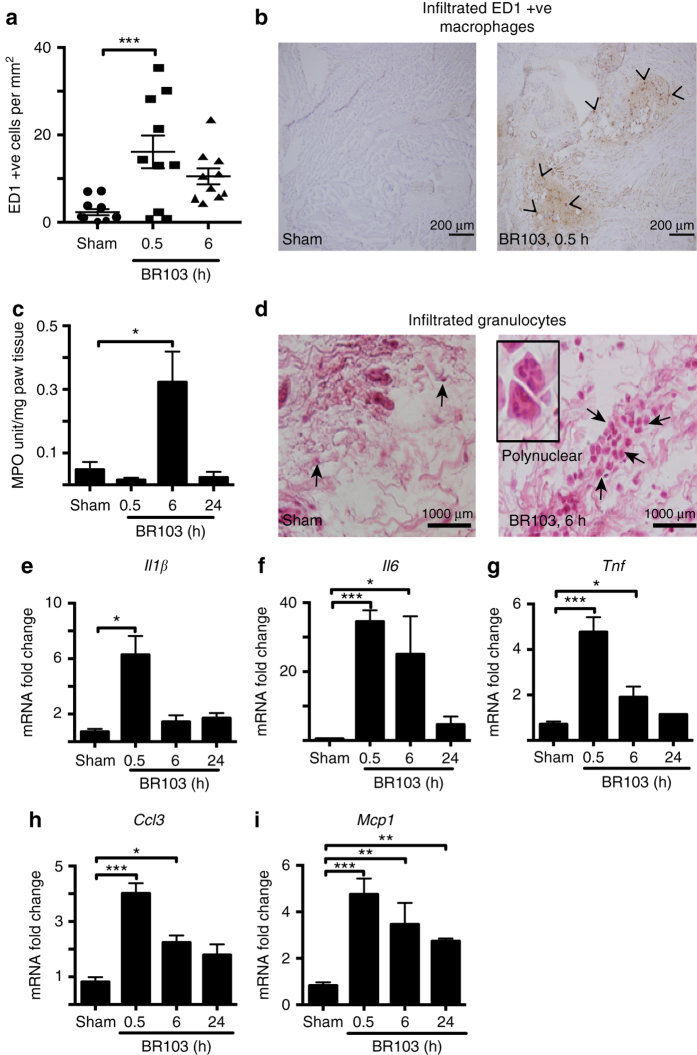
Agonist **3** (BR103) induces temporal inflammatory responses in rat paws. BR103 (compound **3**, 350 µg/paw, i.pl.) induces: **a**, **b** increased infiltration of ED1^+^ macrophages at 30 min but much less at 6 h compared to sham (vehicle) as demonstrated by immunohistochemistry staining of ED1^+^ (*black chevron*), *Scale bar*: 200 μm; **c** increased myeloperoxidase (*MPO*) activity at 6 h, but not at 30 min or 24 h compared to sham (vehicle); **d** infiltrated leukocytes and granulocytes after 6 h compared to sham (vehicle) (*arrowheads* – granulocytes), *Scale bar*: 1000 μm; **e**–**i** upregulation of proinflammatory genes such as *Il1β* **e**, *Il6* **f**, *Tnf* **g**, *Ccl3* **h**, and *Mcp1* **i**, which peaked at 30 min compared to sham (vehicle). *Error bars* represent mean ± SEM. *P < 0.05; **P < 0.01; ***P < 0.005 (one-way ANOVA, Uncorrected Fisher’s LSD post hoc comparison). 8- to 9-week-old male Wistar rats (*n* = 29) were used for this experiment

A single intraplantar injection of the C3aR agonist, BR103 (compound **3**), induced an acute inflammatory edema, similar to that shown by λ-carrageenan and other irritants, with localized paw swelling, mast cell activation, histamine and tryptase release, myeloperoxidase (MPO) activation, leukocyte recruitment and expression of various proinflammatory cytokines in a time-dependent manner **(**Figs. 4a–h and 5a–i **)**. The BR103-induced paw swelling peaked at 0.5 h and returned to baseline after 24 h **(**Fig. 4a **)**. Histological evaluation at the various time points after BR103 administration suggested that resident mast cell activation and degranulation **(**Fig. 4b, c **)** was associated with paw inflammation, which preceded the infiltration and activation of other leukocytes such as inflammatory macrophages and neutrophils. The BR103-induced peak paw swelling at 0.5 h was concurrent with an increased number of activated mast cells, together with increased histamine secretion and decreased tryptase-positive cells in paw tissue compared to vehicle-treated animals **(**Fig. 4d–f **)**. This increase in mast cell activation and degranulation at 0.5 h was a temporal response, since activated mast cells subsided at 6 h and 24 h time points after C3aR activation **(**Fig. 4c **)**.

Interestingly, BR103 also induced a temporal increase in infiltration of inflammatory ED1^+^ macrophages at 0.5 h (Fig. 5a, b) without significantly affecting resident ED2^+^ macrophage cell numbers in paws compared to vehicle-treated animals **(**Fig. 4g, h **)**. Further, BR103 also increased infiltration and activation of other granulocytes and leukocytes such as neutrophils compared to vehicle-treated animals **(**Fig. 5c, d **)**. However, activation of neutrophils (measured as increased activity of MPO) and accumulation of other granulocytes and leukocytes were not observed until 6 h after BR103 administration **(**Fig. 5c,d **)**, suggesting a temporal involvement following recruitment to the site of inflammation.

Moreover, since both immune and non-immune cells have been implicated in C3aR-induced innate immune responses^6^, we further characterized changes in inflammatory gene expression in rat paw tissue at 0.5, 6 and 24 h after BR103 administration **(**Fig. 5e–i **)**. BR103 induced the upregulation of many proinflammatory genes, such as *Il1β*, *Il6*, *Tnf*, *Ccl3*, and *Mcp1*, compared to vehicle-treated animals **(**Fig. 5e–i **)**. Most of these C3aR-induced inflammatory markers are early response genes, peaking at 0.5 h and slowly subsiding over 6 h and 24 h time points after BR103 administration **(**Fig. 5e–i **)**.

### Antagonism of acute edema and innate immune responses

**Fig. 6:**
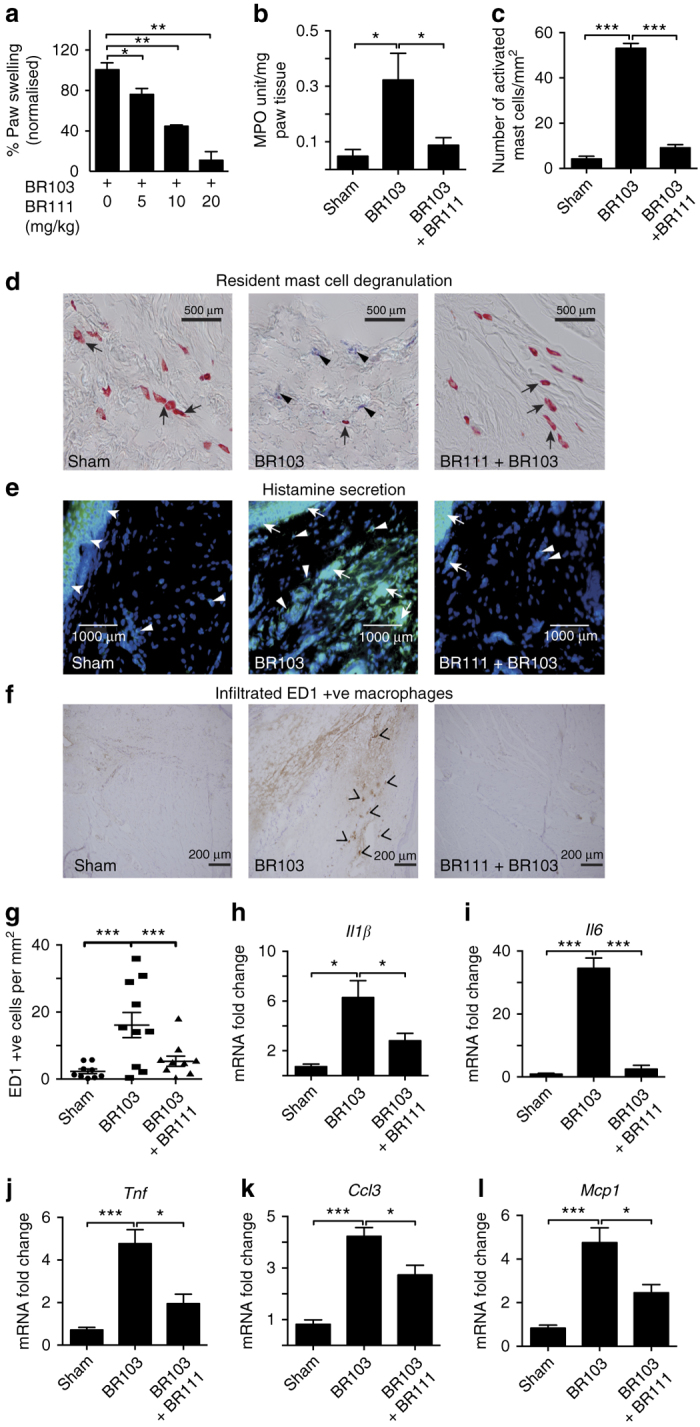
Antagonist BR111 (**6**) inhibits inflammatory responses induced by BR103 (**3**) in rats. Oral pre-treatment with BR111 (**6**, 20 mg/kg p.o., 2 h prior) inhibits BR103 (**3**, 350 µg/paw, i.pl.) induced: **a** paw swelling at 30 min in a dose-dependent manner; **b** increased MPO activity at 6 h; **c**, **d** activated/degranulated mast cells at 30 min stained purplish blue by alcian blue (*arrowheads*), whereas inactive mast cells stained red with safranin O (*arrows*), *Scale bar*: 500 μm; **e** histamine secretion in rat paw tissue at 30 min, as demonstrated by immunohistochemistry staining of histamine, the blue dye stains for the cell nucleus (*white arrowheads*), while the green dye stains for histamine (*white arrows* – diffused extracellular histamine staining), *Scale bar*: 1000 μm; **f**, **g** increased infiltration of ED1^+^ macrophages (*black chevron*) at 30 min as demonstrated by immunohistochemistry staining, *Scale bar*: 200 μm; **h**–**l** expression of inflammatory genes at 30 min *Il1β* **h**, *Il6* **i**, *Tnf* **j**, *Ccl3* **k**, and *Mcp1* **l**. *Error bars* represent mean ± SEM. *P < 0.05; **P < 0.01; ***P < 0.005 (one-way ANOVA, Uncorrected Fisher’s LSD post hoc comparison) 8-to 9-week-old male Wistar rats (*n* = 27) were used for this experiment

Dysregulated C3aR expression and activation have been shown to be involved in various chronic inflammatory disease conditions^4–6^. Verifying that our novel small molecule C3aR antagonists have in vivo functionality would furnish useful therapeutic tools for regulating acute and chronic inflammatory disease conditions. BR111 (compound **6**) was assessed in vivo in the above rat model of acute inflammatory edema induced by C3aR agonist, BR103. When given as an oral pretreatment, BR111 strongly inhibited acute rat paw edema, induced 2 h later by i.pl. injection of BR103 **(**Fig. 6a **)**. Orally administered BR111 inhibited BR103-induced paw swelling at 30 min in a dose-dependent manner, suggesting specificity and competition for C3aR in vivo **(**Fig. 6a **)**. Furthermore, this pre-treatment with BR111 attenuated BR103-induced acute inflammatory responses such as mast cell activation and degranulation **(**Fig. 6c, d **)** and secretion of histamine **(**Fig. 6e **)**, in these rat paws at 0.5 h after agonist administration. BR111 also attenuated neutrophil activation at 6 h after BR103 administration **(**Fig. 6b **)**. BR111 also prevented the increased infiltration of inflammatory ED1 macrophages at 0.5 h in paws compared to BR103-treated animals **(**Fig. 6f, g **)**. These effects were observed together with the suppression of BR103-induced gene expression of proinflammatory cytokines and chemokines by oral BR111 treatment **(**Fig. 6h–l **)**.

These results strongly suggest that the novel C3aR ligand, BR111 (compound **6**), is a potent, selective, orally active antagonist of C3aR with beneficial anti-inflammatory properties in vivo. In summary, the data are consistent with C3aR being activated in vivo by BR103 (compound **3**) and antagonized by BR111 and have helped to characterize for the first time the direct responses of selective C3aR activation and inhibition in vivo on acute innate immune responses.

### Mast cell stabilizer attenuates C3aR inflammation in rats

**Fig. 7:**
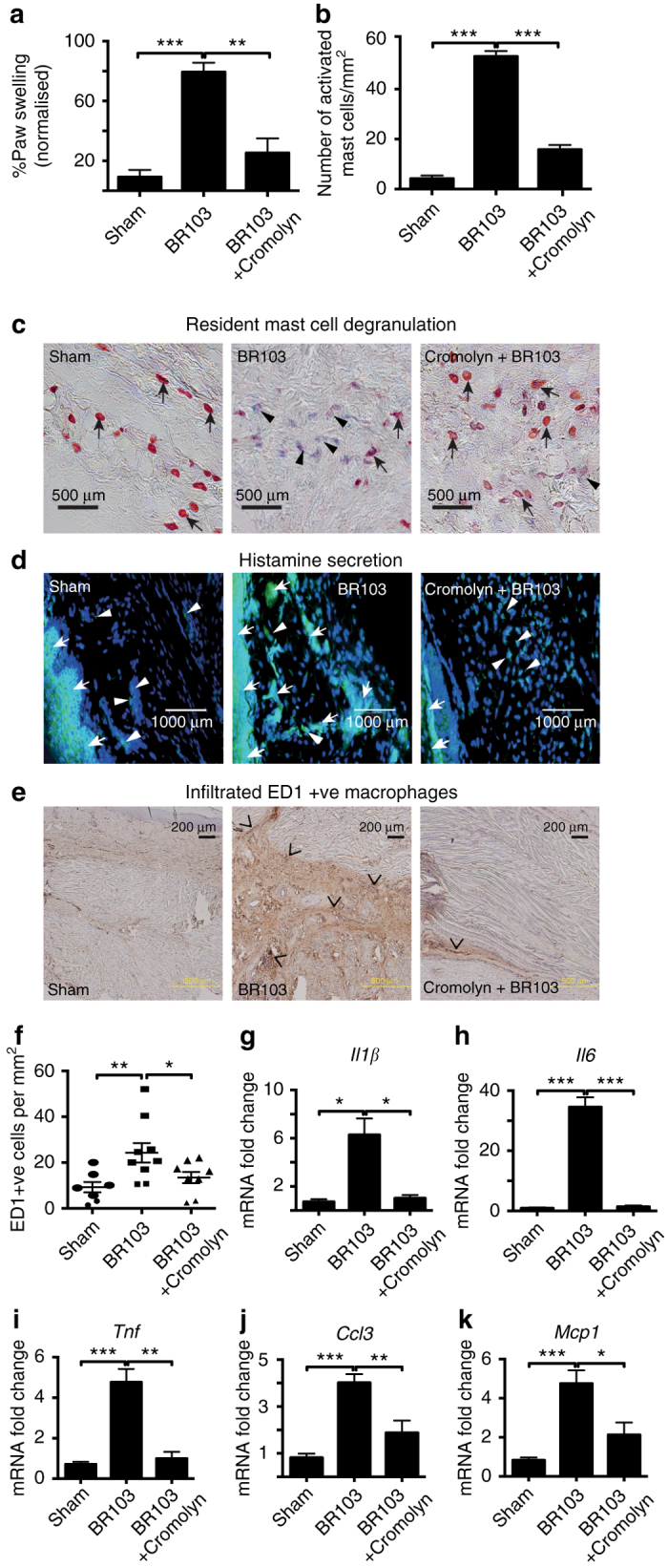
Cromolyn inhibits BR103-induced inflammatory responses in rat paws. Pre-treatment with cromolyn (20 mg/kg s.c., 30 min) inhibits BR103 (compound **3**, 350 µg/paw, i.pl.) induced: **a** paw swelling at 30 min; **b**, **c** activated/degranulated mast cells at 30 min stained purplish blue by alcian blue (*arrowheads*), whereas inactive mast cells stained red with safranin O (*arrows*), *Scale bar*: 500 μm; **d** histamine release in rat paw tissue at 30 min, as demonstrated by immunohistochemistry staining of histamine, the blue dye stains for the cell nucleus (*white arrowheads*), while the green dye stains for histamine (*white arrows* – diffused extracellular histamine staining), *Scale bar*: 1000 μm; **e**, **f** increased infiltration of ED1^+^ macrophages (*black chevron*) at 30 min as demonstrated by immunohistochemistry staining, *Scale bar*: 200 μm; **g**–**k** inflammatory gene expression at 30 min *Il1β* **g**, *Il6* **h**, *Tnf* **i**, C*cl3* **j**, and *Mcp1* **k**; *Error bars* represent mean ± SEM. *P < 0.05; **P < 0.01; ***P < 0.005 (one-way ANOVA, Uncorrected Fisher’s LSD post-hoc comparison). 8- to 9–week-old male Wistar rats (*n* = 27) were used for this experiment

Finally, we investigated whether the C3aR agonist-induced acute inflammatory edema and immune response in vivo was specifically mediated by a mast cell-dependent mechanism or whether resident or infiltrating macrophages or neutrophils were contributors. Resident mast cells are generally localized at peripheral sites, including the skin, airways, gut, and other tissues. Rapid activation of mast cells has been shown to initiate inflammation through the release of both pre-formed and synthesized mediators, which induce increased vasopermeability, vasodilation, accumulation of inflammatory mediators, and chemotaxis of other immune cells^28–30^. Thus, to further characterize mechanistically if BR103-induced acute inflammatory edema in rats was mostly mediated via mast cells, we investigated the pharmacological responses of a known mast cell stabilizer, cromolyn, on inflammation induced by BR103 (compound **3**). Cromolyn is believed to prevent mast cell degranulation by stabilizing the cell membrane and it is currently used to treat various mast cell related diseases such as asthma, conjunctivitis, allergic rhinitis, and mastocytosis^31^. When rats were pretreated with cromolyn (20 mg/kg s.c., 30 min prior) there was a significant reduction in BR103-induced paw swelling at 0.5 h, almost back to baseline levels **(**Fig. 7a **)**. Cromolyn treatment in vivo also attenuated BR103-induced increased activation **(**Fig. 7b **)** and degranulation **(**Fig. 7c **)** of mast cells together with reduced histamine expression **(**Fig. 7d **)** at 0.5 h in rat paws. This was followed by a reduced infiltration of inflammatory ED1^+^ macrophages at 0.5 h in rat paws compared to BR103-treated animals **(**Fig. 7e, f **)** and a reduced expression of early response inflammatory genes, including *Il1β*, *Il6*, *Tnf*, *Ccl3*, and *Mcp1* at 0.5 h **(**Fig. 7g–k **)**. Taken together, these findings indicate that the direct activation of C3aR in vivo by BR103 (compound **3**) results in acute inflammatory edema and innate immune responses that are specifically initiated via a mast cell-dependent mechanism. Thus, C3aR on resident mast cells clearly has a key initiating and amplifying role in acute events such as degranulation, histamine and tryptase secretion, leading to paw inflammation, cytokine expression and other associated innate immune responses in vivo. Other key innate immune cells such as infiltrating macrophages and neutrophils are subsequently recruited to the site of inflammation and can drive a sustained immune response, but they do not seem to be major contributors to the early acute innate immune response induced in vivo in rats through C3aR activation.

## Discussion

Human C3a (hC3a) is one of ~ 30 complement proteins produced in blood or on cell surfaces through activation by infection or injury^4–6^. Collectively, they “complement” immune cells and antibodies to identify, tag, destroy, and eliminate pathogens and infected or damaged cells, and repair wounds^4–6^. If the inflammatory stimulus is not removed, complement activation becomes prolonged, or misdirected to healthy cells, causing or exacerbating chronic inflammatory or auto-immune diseases. Multiple pathways of complement activation produce C3a, which is thought to stimulate important immune and metabolic responses. Its human receptor, hC3aR is considered to be a key mediator of inflammatory disease^4–6^, but current small molecule ligands for hC3aR are not sufficiently drug-like to be effective probes for in vivo studies. Development of potent, selective, metabolically stable and bioavailable ligands for C3aR could be valuable new leads to a new class of therapies for treating complement-mediated diseases.

Here we describe different heterocycles adjacent to a carboxamide that can be used to control ligand conformation, as established by 2D NMR spectroscopic techniques. We have demonstrated how this conformational change, triggered by a simple change of heteroatom, profoundly impacts on ligand function, resulting in potent agonists (e.g., **3**, BR103) or antagonists (e.g., **6**, BR111). This chemical discovery has been then exploited to unravel new cell and in vivo biology, by examining the effects of potent and selective small molecule agonists that mimic C3a in activating mast cells, macrophages and neutrophils, and of potent small molecule antagonists that block the actions of both C3a and BR103 on these innate immune cells. These small molecule modulators were selective for C3aR, showing functional responses on human LAD2 mast cells, HMDM cells, and HEK293 cells that expressed C3aR but not on C3aR^−/−^ HEK293 cells. They were also selective for C3aR over C5aR, and were metabolically stable chemical tools for use in animal models.

Direct administration of a potent C3aR agonist intraplantar to rat paws elicited an acute edema and innate immune response, characterized by mast cell activation and degranulation, histamine and tryptase release, expression of inflammatory cytokines (IL-1β, CCL3, IL6, TNF, MCP1), and macrophage and neutrophil infiltration. All of these measures of acute inflammatory responses were inhibited by oral pretreatment of a potent C3aR antagonist delivered systemically to rats. An important mechanistic finding was that the mast cell stabilizer cromolyn, which blocks degranulation of mast cells but does not inhibit neutrophil or macrophage functions, was also able to inhibit agonist-induced paw inflammation in rats. C3a and its small molecule agonist mimics are able to activate a range of innate immune cells such as mast cells, macrophages and neutrophils. However, these results support the conclusion that it is the mast cell that initiates the acute innate immune response and that other immune cells that infiltrate damaged or infected tissues subsequently drive inflammation.

Mast cells have been almost the forgotten innate immune cells until recently. They were identified over 100 years ago, are conserved across many species, yet their roles in immunity, defence and disease are still not completely defined^30–32^. They are special in containing as many as 500,000 secretory granules^32^, filled with numerous pre-formed and pre-activated immunomodulators^30–32^ that are released upon degranulation, but are also known to secrete inflammatory mediators in the absence of degranulation^30, 32^. Although mast cells have been regarded as sentinels for infection and drivers of allergic and anaphylactic responses through IgE^28, 33^, they are also emerging as potentially important contributors to chronic inflammatory diseases such as inflammatory bowel disease, irritable bowel syndrome, multiple sclerosis, arthritis, wound healing, graft rejection, angiogenesis, cancers, cardiovascular diseases, diabetes, obesity, and others^28^. Recent single-cell transcriptomic studies have identified new progenitor populations with mast-cell potential that are distinct from the neutrophil-monocyte lineage and that segregate early in hematopoietic development^34^. Transcriptomic analysis in isolated constitutive connective-tissue mast cells from skin, tongue, oesophagus, trachea, and the peritoneal cavity have identified distinct gene clusters for mast cells independent of other lymphoid and myeloid-cell populations^30, 35^. These studies are revealing previously unappreciated mast-cell turnover rates and pre-activated secretory granule turnover in the periphery in the absence of tissue inflammation^30, 35^. Human C3a is known to degranulate mast cells in vitro to release histamine and tryptase^21^, but evidence for the action of C3a in vivo on mast cells remains clouded. Our data for C3aR activation in rat paws by an agonist and blockade by an antagonist suggests that C3a is proinflammatory in vivo through its action on resident mast cells in the skin, and that mast cell degranulation is a key initiator of acute inflammation in the paw and a stimulator of recruitment of other immune cells (macrophages, neutrophils) that are subsequently activated. Further studies using our novel ligands may help identify specific roles of mast cells in other distinct anatomical locations in health and disease, and in more chronic inflammatory diseases where mast cell turnover could be important in sustaining an inflammatory stimulus.

Historically, C3a has been thought to be formed in the liver through proteolytic cleavage of serum complement C3^36^, but it is now emerging that many cell types, including cells of myeloid, lymphoid, and non-myeloid, non-lymphoid origin can generate C3a from transported C3 presented at the cell surface where C3 convertases are formed^36^. For example, activation of T cells induces the local expression of C3, factor B and factor D that results in the extracellular assembly of C3 convertase and cleavage to C3a that acts on the cell surface C3aR^37^. Rapid cleavage of the C-terminal Arg of C3a to C3a-desArg by carboxypeptidases may be an efficient defence mechanism to dampen an inflammatory response and restrict the action of C3a to the cell surface. Availability of the first truly potent, stable, selective, and bioavailable C3a agonists and antagonists reported here could be valuable new molecular tools for interrogating the sequence of cellular immune responses to C3aR activation in vivo in different pathological settings.

This report is the culmination of attempts to downsize the 77-residue complement C3a protein to potent and selective small molecule modulators that are stable enough to be used in vivo as chemical tools to probe C3a-dependent biology. Unlike many protein–protein interactions, the bioactive effector region of C3a is localized to a small region around its C-terminal arginine. However, the C-terminal tetrapeptide segment of C3a alone has no detectable C3aR activity and it has been challenging to obtain a small molecule agonist with equal potency and functional selectivity as the C3a protein. Further, the rational development of a potent and C3aR-selective antagonist suitable for use in vivo was also a challenging goal. Here we have shown how to use different heterocycles to produce potent and selective C3aR ligands, exploiting a novel conformational switch by positioning the heterocycle adjacent to a carboxamide to change agonists into antagonists. This resulted in potent and selective agonists and antagonists that are very effective inflammatory modulators and valuable chemical biology tools when administered to rats. This approach to potent small molecule protein mimics exemplified here for one protein, might also be more generally applicable and transferable to other protein–protein interactions, where site-directed mutagenesis has identified hot spots responsible for protein function. It remains to be determined whether this approach also leads to small molecules with similarly potent and selective functional activities and selectivity for other protein targets.

### HPLC studies

Preparative-scale reversed-phase HPLC (rpHPLC) separations were performed on a Phenomenex Luna C18 10 μm, 250 × 30.0 mm column. Standard conditions were used for elution of all compounds: 100% A to 100% B linear gradient over 20 min, followed by a further 10 min at 100% B, where solvent B was 90% MeCN, 10% H_2_O + 0.1% TFA, and solvent A was H_2_O + 0.1% TFA, at a flow rate of 40 mL min^−1^. Compounds were detected by UV spectroscopy and pure fractions were lyophilized. Analytical rpHPLC was used to assess compound purity (Phenomenex Luna C18 column, 5 μm, 90 Å, 4.6 × 250 mm, at three different wavelengths *λ* = 214, 230 and 254 nm). Standard conditions (same as preparative-scale rpHPLC) were used for all compounds at a flow rate of 1 mL min^−1^. All final compounds were ≥ 95% pure by analytical HPLC.

### LCMS studies

Electrospray ionization mass spectral measurements were obtained on a Micromass LCT spectrometer. High-resolution mass spectra (HRMS) measurements were obtained on a Bruker microTOF mass spectrometer equipped with a Dionex LC system (Chromeleon) in positive ion mode by direct infusion in MeCN at 100 μL h^−1^ using sodium formate clusters as an internal calibrant. The data were processed using Bruker Daltonics DataAnalysis 3.4 software. Mass accuracy was consistently better than ± 1 ppm.

### NMR studies

^1^H and ^13^C NMR spectra were recorded on Bruker Avance III HD 600 equipped with a cryoprobe or Varian 400 spectrometers at 298 K using deuterated solvents and were referenced to the residual solvent signal DMSO-d_6_^1^H 2.50 ppm, ^13^C 39.51 ppm.  CDCl_3_ solutions were referenced to internal tetramethylsilane (TMS). The exact concentration of the compound for assay was confirmed by the quantitative NMR integration ‘PULCON’ experiment. Settings for all PULCON experiments: relaxation delay *d1* = 30 s, 16 scans, 2 dummy scans, 90° pulse and temperature at 298 K. ROESY spectra were acquired using the standard Bruker pulse sequence roesyph with a spinlock pulse of 350 ms and relaxation delay 2 s.

### Reagents

All reagents were purchased from Sigma-Aldrich or Chem-Impex International Inc. C3a-desArg (cat. No. C3A16-N-25) was purchased from Jomar Life Research, Australia. All compounds were synthesized via solid- or solution-phase chemistry approaches. The C5a receptor antagonist (3D53, also called PMX53)^16^ is Ac-cyclo-(2,6)-Phe-[Orn-Pro-dCha-Trp-Arg], synthesized by Boc chemistry on a 0.20 mmol scale using HBTU/DIPEA activation and in situ neutralization on Boc-l-Arg(Tos)-4-hydroxymethyl-phenylacetamidomethyl resin (PAM resin), cleavage and deprotection with HF and *p*-cresol (1 ml), cyclization with BOP and DIPEA in DMF^38^, or it was synthesized in solution as described^39^.

### Spectroscopic data for the characterisation of compounds 1–6

Supplementary Figs. 1–5 outline the syntheses of all compounds and full experimental details are available in the Supplementary Methods. Proton and carbon NMR spectra are shown for compounds **1**–**6** in Supplementary Figs. 6–17. The proton and carbon NMR data recorded on the instruments above, and the HRMS data and retention times (HPLC *t*_R_) measured under standard HPLC conditions, for all compounds used in this study are catalogued below.

*Compound* ***1.*** ^1^H NMR (600 MHz, DMSO-d_6_): δ 8.28 (d, *J* = 8.0 Hz, 1H), 8.23 (s, 1H), 7.50 (t, *J* = 5.3 Hz, 1H), 7.37–7.27 (m, 10H), 6.03 (s, 1H), 4.42–4.39 (m, 1H), 3.11–3.07 (m, 2H), 1.90–1.83 (m, 1H), 1.81–1.74 (m, 1H), 1.51–1.45 (m, 2H). ^13^C NMR (150 MHz, DMSO-d_6_): δ 173.5, 173.0, 160.4, 156.6, 149.5, 141.8, 128.7, 128.67, 128.66, 127.3, 127.2, 124.9, 53.6, 51.6, 40.3, 28.0, 25.3. HRMS calculated for C_23_H_26_N_5_O_3_S^+^ 452.1751, found 452.1750. HPLC *t*_R_ 14.6 min.

*Compound* ***2.*** ^1^H NMR (600 MHz, DMSO-d_6_): δ 8.14 (d, *J* = 8.0 Hz, 1H), 7.54 (t, *J* = 5.7 Hz, 1H), 7.39–7.33 (m, 4H), 7.33–7.25 (m, 6H), 5.94 (s, 1H), 4.39 (m, 1H), 3.14–3.06 (m, 2H), 2.68 (s, 3H), 1.86 (m, 1H), 1.76 (m, 1H), 1.53–1.43 (m, 2H). ^13^C NMR (150 MHz, DMSO-d_6_): δ 173.1, 168.4, 161.8, 156.6, 142.3, 141.8, 140.8, 128.7, 128.6, 127.2, 53.5, 51.3, 40.3, 28.2, 25.3, 12.2. HRMS calculated for C_24_H_28_N_5_O_3_S^+^ 466.1907, found 466.1907. HPLC *t*_R_ 15.4 min.

*Compound* ***3.*** *(BR103)* ^1^H NMR (600 MHz, DMSO-d_6_): δ 7.91 (d, *J* = 7.5 Hz, 1H), 7.65 (t, *J* = 5.6 Hz, 1H), 7.37–7.32 (m, 4H), 7.30–7.24 (m, 6H), 5.69 (s, 1H), 4.38 (m, 1H), 3.16–3.05 (m, 2H), 2.41 (s, 3H), 1.83 (m, 1H), 1.72 (m, 1H), 1.55–1.46 (m, 2H). ^13^C NMR (150 MHz, DMSO-d_6_): δ 173.4, 161.7, 156.8, 146.7, 140.3, 132.4, 128.7, 128.6, 127.1, 51.1, 49.4, 40.3, 28.5, 25.3, 10.6. HRMS calculated for C_24_H_29_N_6_O_3_^+^ 449.2296, found 449.2295. HPLC *t*_R_ 11.5 min.

*Compound* ***4.*** ^1^H NMR (600 MHz, DMSO-d_6_): δ 8.84 (d, 1H, *J* = 7.8 Hz), 8.43 (s, 1H), 7.61 (t, 1H, *J* = 5.5 Hz), 7.29–7.37 (m, 10H), 6.00 (s, 1H), 4.32–4.35 (m, 1H), 3.08–3.15 (m, 2H), 1.82–1.88 (m, 1H), 1.67–1.73 (m, 1H), 1.49–1.59 (m, 2H). ^13^C NMR (150 MHz, DMSO-d_6_): δ 176.9, 173.2, 160.0, 156.6, 143.9, 141.7, 134.6, 128.71, 128.67, 127.2, 53.8, 52.0, 40.3, 27.7, 25.4. HRMS: [MH]^+^ calc. for C_23_H_26_N_5_O_3_S^+^ 452.1751, found 452.1751. HPLC *t*_R_ 14.2 min.

*Compound* ***5.*** ^1^H NMR (600 MHz, DMSO-d_6_): δ 8.41 (d, *J* = 7.8 Hz, 1H), 7.52 (t, *J* = 5.6 Hz, 1H), 7.39–7.26 (m, 10H), 5.94 (s, 1H), 4.29 (m, 1H), 3.13–3.04 (m, 2H), 2.52 (s, 3H), 1.81 (m, 1H), 1.67 (m, 1H), 1.57–1.44 (m, 2H). ^13^C NMR (150 MHz, DMSO-d_6_): δ 173.1, 172.6, 161.4, 156.6, 154.9, 141.7, 128.7, 127.2, 125.5, 53.7, 52.2, 40.3, 27.5, 25.4, 17.0. HRMS calculated for C_24_H_28_N_5_O_3_S^+^ 466.1907, found 466.1907. HPLC *t*_R_ 14.3 min.

*Compound* ***6.*** (*BR111*) ^1^H NMR (600 MHz, DMSO-d_6_): δ 8.60 (d, *J* = 8.0 Hz, 1H), 7.73 (d, *J* = 3.8 Hz, 1H), 7.58 (t, *J* = 5.5 Hz, 1H), 7.37–7.22 (m, 11H), 6.79 (dd, *J* = 3.8, 1.0 Hz, 1H), 5.83 (s, 1H), 4.32 (m, 1H), 3.16–3.04 (m, 2H), 1.85 (m, 1H), 1.70 (m, 1H), 1.61–1.46 (m, 2H). ^13^C NMR (150 MHz, DMSO-d_6_): δ 173.4, 161.2, 156.7, 153.0, 143.2, 137.8, 128.6, 128.5, 126.9, 51.9, 51.2, 40.3, 27.7, 25.4. HRMS calculated for C_24_H_27_N_4_O_3_S^+^ 451.1798, found 451.1798. HPLC *t*_R_ 15.2 min.

### LAD2 mast cell culture and histamine assay

LAD2 human mast cells (kindly provided by Dr Dean Metcalfe, National Institute of Allergy and Infectious Diseases, National Institute of Health) were cultured in StemPro^®^-34 Serum Free Media (Invitrogen, Australia) supplemented with StemPro^®^-34 nutrient, 100 U/mL penicillin, 100 μg/mL streptomycin, 2 mM GlutaMAX, and 100 ng/mL human stem cell factor (SCF). Cells were hemi-depleted weekly and were not allowed to grow beyond a density of 0.5 × 10^6^/mL. This cell line was authenticated by expression of tryptase and histamine granules. For the histamine assay, 1 × 10^4^ cells per well were seeded in StemPro^®^-34 SFM in 96-well plates for 30 min before any treatments. For agonist studies, the cells were treated with compounds prepared in media for 30 min. For antagonist studies, the cells were pre-treated with compounds prepared in SFM for 30 min before addition of C3aR agonists for another 30 min. Cells were centrifuged (500 g, 5 min, 4 °C) and cell-free supernatants were collected for measuring histamine. Histamine content was measured using the histamine EIA kit according to the manufacturer’s instructions (Abnova, Taiwan). The readings were recorded on a FLUOstar Optima (BMG LabTechnologies, Offenburg, Germany).

### Isolation of primary human monocyte-derived macrophages

HMDMs were isolated using Ficoll-paque density centrifugation (GE Healthcare Bio-Science, Uppsala, Sweden) from buffy coat (of anonymous human donors) provided by the Australian Red Cross Blood Service, Brisbane. CD14^+^ monocytes were positively selected using CD14 MicroBeads (Miltenyi Biotech, Auburn, USA)^40, 41^. CD14^+^ monocytes were differentiated to HMDMs using 100 ng/mL of recombinant human macrophage colony-stimulating factor (PeptroTech, Rocky Hill, USA) for 7 days in Iscove’s modified Dulbecco’s media supplemented with 10% fetal bovine serum, penicillin (100 U/mL), streptomycin (100 µg/mL) and L-glutamine (2 mM). HMDMs were supplemented after 5 days with fresh medium containing 10 ng/ml macrophage colony-stimulating factor and collected by gentle scraping on day 7.

### Intracellular calcium release assay

HMDMs were seeded to a 96-well clear-bottom black-wall assay plate at a density of 5 × 10^4^ cells per well and allowed to adhere overnight. Prior to assay, cells were incubated with dye-loading buffer (Hank’s balance salt solution (HBSS) buffer, 4 μM Fluo-3 AM, 25 μl Pluronic acid F-127 and 1% fetal bovine serum) for 1 h at 37 °C. Cells were then washed once with assay buffer (HBSS supplemented with 2.5 mM probenecid and 20 mM HEPES, pH 7.4). Intracellular calcium release was monitored using a fluorescence imaging plate reader (excitation 495 nm, emission 520 nm). The percentage responses were plotted against logarithmic concentrations of each test compound^42^.

### ^125^I-C3a and ^125^I-C5a radioligand binding assay

Receptor binding was performed by ligand competition with labelled 80 pM [^125^I]-C3a or 25 pM [^125^I]-C5a (2200 Ci/mmol; Perkin Elmer, Torrance, CA, USA) on HMDMs (1.2 × 10^6^ cells/ml). HMDMs were treated with [^125^I]-C3a or [^125^I]-C5a ± various concentrations of unlabelled C3a/C5a or experimental ligands were diluted with assay buffer (50 mM Tris, 3 mM MgCl_2_, 0.1 mM CaCl_2_, 0.5% (w/v) BSA, pH 7.4) for 60 min at room temperature. Unbound [^125^I]-C3a or [^125^I]-C5a was removed by filtration through glass microfibre filter GF/B (Whatman Iner. Ltd, England) and washed three times with cold buffer (50 mM Tris-HCl pH 7.4). Bound [^125^I]-C3a or [^125^I]-C5a were assessed by scintillation counting on Microbeta counter. Specific binding was defined as the difference between total binding and nonspecific binding as determined in the presence of 1 μM unlabelled C3a or C5a^4^.

### Europium (Eu)-C3a competitive binding assay

HEK293 Gα_16_-C3aR cells were non-enzymatically lifted using Versene Solution and seeded at 30,000 cells per well in 2% BSA in phosphate buffered saline (PBS) in a round-bottom 96-well plate. Cells were simultaneously treated with Eu-DTPA-C3a (2 nM) and various concentrations of C3a or C3a-desArg for 60 min at room temperature with shaking. Cells were then washed thrice with PBS supplemented with 0.2% BSA, 20 µM EDTA and 0.01% Tween-20 by repeated centrifugation. Cells were then resuspended with 20 µL of DELFIA enhancement solution (PerkinElmer) and then transferred to a white 384-well ProxiPlate (PerkinElmer). Time-resolved fluorescence was measured using a PHERAstar plate reader (BMG Labtech) at 337 nm excitation followed by 400 µs delay before 620 nm emission.

### Immunobloting

Human Embryonic Kidney (HEK) 293 cells (ATCC, USA) and HMDMs were lysed on ice. Equal amounts of cell lysates were separated, transferred using iBlot Dry Blotting System (Invitrogen). Human C3aR was detected with mouse monoclonal IgG_2a_ (Santa Cruz Biotechnology, SC-133172, 1:200) and secondary HRP-linked anti-mouse IgG (Cell Signaling Technology, #7076, 1:500). GAPDH was detected using affinity isolated rabbit anti-GAPDH (Sigma-Aldrich, G9545, 1:10,000) and secondary HRP-linked anti-rabbit IgG (Cell Signaling Technology, #7074, 1:10,000). An example of a full blot is shown in Supplementary Fig. 18.

### Plasma and metabolic stability

For plasma stability studies, C3a and experimental compounds (10 μL, 1 mM in DMSO) were added to neat rat plasma (190 μL) and heated to 37 °C in a circulating water bath. Aliquots (20 µL) were collected at time points (0, 2, 5 and 10 min for C3a; 0, 5, 15, 30, 60, 120 and 240 min for other test compounds) and added directly to MeCN/H_2_O (80 µL, 9:1). The samples were centrifuged at 13,000 g for 3 min before supernatants were isolated and analyzed for test compound degradation via LC-MS. For liver microsomal stability studies, commercially available rat liver microsomes from Life Technologies, GIBCO were used. In brief, microsomes stored at −80 °C were slowly defrosted on ice. Test compounds (183 μL, 1 μM in 100 mM phosphate buffer pH 7.4 containing 0.1 % DMSO) were added to microsomes (5 μL) and NADPH (2 µL, 20 mM). Test solutions were heated in a circulating water bath at 37 **°**C for 5 min before additional cofactor NADPH (10 μL, 20 mM) was added. Aliquots (20 μL) were taken at time points (0, 5, 15, 30 and 60 min from the second addition of NADPH). Aliquots were added to MeCN/H_2_O (80 μL, 9:1) and centrifuged at 13,000 g for 3 min. Supernatants were analyzed for test compound degradation by LC-MSMS. Concentrations were plotted as a function of time on a semi-logarithmic graph. Rat plasma and microsomal activity was confirmed by rapid degradation of SLIGRL-NH_2_ (t_1/2_ < 5 min) in a parallel experiment.

### Animals

All experiments were approved by the Molecular Biosciences Animal Ethics Committee of The University of Queensland, and adhere to The Australian Code of Practice for Use Of Animals for Scientific Purposes (2013) and The Australian Government Guidelines to Promote the Wellbeing of Animals used For Scientific Purposes (2013). Studies involving animals are reported in accordance with the ARRIVE guidelines^43^. A total of 109 animals were used in the experiments described here. Numbers were determined using power analysis (Effect size; 0.25, Power 0.8. G*Power 3.1.9) Male Wistar rats (8–9 weeks, 250 ± 20 g) were bred at the Australia Animal Resource Centre (Canning Vale, WA, Australia). Animals were housed in the appropriate temperature/pressure environment in a 12 h light/dark cycle, according to the standards of the accredited holding facility, with food and water provided ad libitum. At least 48 h habituation in the UQBR facility was provided prior to any experimental intervention. After experimentation, animals were humanely killed by CO_2_ inhalation as stipulated by approved ethical agreements.

### Rat paw edema

Male Wistar rats (8–9 weeks) were injected with BR103 (**3**, 350 μg in 100 μL isotonic saline) into the plantar surface of both hind paw pads using a 30G needle. The inhibitor cromolyn (20 mg/kg in 5% DMSO) was injected subcutaneously 30 min before the agonist. The antagonist BR111 (**6**, 5–20 mg/kg in olive oil) was administered orally as a single dose 2 h before the agonist. Paw thickness and width were measured using digital calipers (WPI) at 0, 0.5, 2, 6 and 24 h after agonist BR103 administration. Hind paw size is expressed as % change in area from baseline after 0.5 h and then normalized against maximum swelling induced by agonist alone. Researchers were not blinded to treatments given.

### Histopathology and immunohistochemistry

Rat paw tissue was collected and fixed in 4% paraformaldehyde (pH 7.4) for 2 h in the cold room (4 °C). The rat paw tissue was then transferred into falcon tubes containing 30% sucrose solution in PBS and left overnight in the cold room. The next day, rat paw tissue was embedded with the Optimal Cutting Temperature compound (OCT, Sakura Finetek, USA) and stored in the −80 °C freezer before being used for making cryo-sections for histological analysis. Frozen sections of 5 microns were prepared using a cryostat-microtome (Leica Biosystems, Germany) and stained using standard protocols on the same day. In normal histological staining, such as with alcian blue and safranin O and hematoxylin and eosin (H&E), samples were briefly washed with distilled water before staining. In alcian blue and safranin O, samples were stained with 0.1% alcian blue (Sigma-Aldrich, USA) and 0.5% safranin O (Sigma-Aldrich, USA) before being mounted in mounting medium. In H&E, samples were stained with hematoxylin (Sigma-Aldrich, USA) and 0.1% eosin (Sigma-Aldrich, USA) before being mounted in mounting medium. In immunohistochemistry, samples were briefly rinsed with PBS to remove OCT and incubated with blocking medium (PBS, 0.1% triton X-100, 10% horse serum) for 1 h at room temperature. Samples were incubated with primary antibody medium (PBS, 4% horse serum, 1:200 histamine or 1:200 of tryptase or 1:150 ED1 or 1:200 ED2 antibodies) overnight at 4 °C. The next day, samples were washed with PBS and were incubated with the secondary antibody medium (PBS, 4% horse serum, detection antibody 1:1000) for 1 h at room temperature. The samples were briefly rinsed, dried and counterstained with DAPI (Invitrogen, Australia). Primary antibodies for histamine (Sigma-Aldrich, USA, H2403, monoclonal rabbit anti-rat IgG, 1: 200), tryptase (Abcam, Australia, ab2378, monoclonal mouse anti-rat IgG, 1:200), ED1 (Serotec, UK, MCA341R, monoclonal mouse anti-rat, 1:150), ED2 (Serotec, UK, MCA3424R, monoclonal mouse anti-rat, 1:200) were purchased from commercial sources. Secondary detection antibody was purchased from (Invitrogen, Australia). ED1 & ED2 positive cells were quantified using FIJI/ImageJ 1.42q software, U.S. National Institutes of Health, Bethesda, MD, USA^44^. All microscope images were obtained using an Olympus BX-51 upright microscope with Olympus DP-71 12Mp colour camera, utilizing DP Capture and DP Manager software packages (Olympus, Tokyo, Japan). Researchers who performed histological analysis and scoring were blinded to the sample identity.

### Myeloperoxidase assay

Rat paw tissues were collected and immediately frozen with liquid nitrogen. The frozen rat paw tissue samples were weighed and crushed into smaller pieces with a mortar and pestle in liquid nitrogen. The crushed tissues were added to MPO buffer (50 mM potassium phosphate buffer, pH 6.0) to make up a concentration of 200 mg/mL and homogenized using Zirconium oxide beads (Next Advance, USA) and the Bullet Blender® homogeniser (Next Advance, USA). The homogenized tissues were then diluted to 100 mg/mL with the MPO buffer containing 1% hexadecyl trimethylamonium bromide (Sigma-Aldrich, USA) and underwent three cycles of freeze, thaw, and sonication. The samples were centrifuged at 13,000 g for 10 min at 4 °C and the clear supernatant for each samples were collected. To measure the MPO release, 10 μL of the sample supernatant was added with 290 μL of substrate solution (MPO buffer, 0.167 mg/mL o-dianisidine.HCl, 0.001% H_2_O_2_) in a clear 96-well plate. The plate was read immediately at 460 nm every min for 20 min using a plate reader (PHERAstar FS, BMG Labtech, Germany). O-dianisidine HCl was purchased from Sigma-Aldrich (USA).

### RNA isolation and gene analysis

Rat paw tissues were collected and immediately frozen with liquid nitrogen. Frozen rat paw tissues were cut into smaller pieces and mixed with 1 mL of TRIsure (Bioline, Australia) prior to homogenizing according to manufacturer’s instructions. Homogenized tissue samples were added with 200 μL of chloroform and mixed thoroughly. The samples were centrifuged at 11,000 g for 15 min. The top clear solution for each sample was collected and isolated using the ISOLATE II RNA Mini Kit (Bioline, Australia) according to the manufacturer’s instructions. RNA was converted to cDNA using SuperScript^®^ III Reverse Transcriptase (Invitrogen, Australia) and Oligo(dT)12-18 primer (Invitrogen, Australia) according to manufacturer’s instructions. Real-time PCR was measured on a ABI PRISM 7900HT (Applied Biosystems), each target gene was normalized to housekeeping 18S rRNA and fold change was calculated relative to control sample (Sham). All samples were done in duplicates. Primer sequences (Supplementary Table 1) were designed using the Primer-Blast online-based software.

### Statistical analysis

All experimental results are expressed as means ± standard error. The data were plotted and analyzed using GraphPad Prism version 5.0c for Mac OS X (GraphPad Software). Statistically significant differences were assessed either using student’s *t*-test for paired comparison or a two-way repeated measures ANOVA as appropriate. All values of independent parameters are shown as mean ± SEM of at least three independent experiments, unless otherwise stated. Significance was set at *P < 0.05, **P < 0.01 and ***P < 0.001.

### Data availability

The data that support the findings of this study are available from the corresponding author upon reasonable request.

## Electronic supplementary material

Supplementary Information

## References

[1] Scott DE, Bayly AR, Abell C, Skidmore J (2016). Small molecules, big targets: drug discovery faces the protein-protein interaction challenge. Nat. Rev. Drug. Discov..

[2] Modell AE, Blosser SL, Arora PS (2016). Systematic targeting of protein-protein interactions. Trends. Pharmacol. Sci..

[3] Mullard A (2012). Protein-protein interaction inhibitors get into the groove. Nat. Rev. Drug Discov..

[4] Reid RC (2013). Downsizing a human inflammatory protein to a small molecule with equal potency and functionality. Nat. Commun..

[5] Masters SL, Simon A, Aksentijevich I, Kastner DL (2009). Horror autoinflammaticus: the molecular pathophysiology of autoinflammatory disease (*). Annu. Rev. Immunol..

[6] Zipfel PF, Skerka C (2009). Complement regulators and inhibitory proteins. Nat. Rev. Immunol..

[7] Strainic MG, Shevach EM, An F, Lin F, Medof ME (2013). Absence of signaling into CD4(+) cells via C3aR and C5aR enables autoinductive TGF-beta1 signaling and induction of Foxp3(+) regulatory T cells. Nat. Immunol..

[8] Proctor LM (2009). Complement factors C3a and C5a have distinct hemodynamic effects in the rat. Int. Immunopharmacol..

[9] Gerard NP, Gerard C (2002). Complement in allergy and asthma. Curr. Opin. Immunol..

[10] Mizutani N, Nabe T, Yoshino S (2009). Complement C3a regulates late asthmatic response and airway hyperresponsiveness in mice. J. Immunol..

[11] Hutamekalin P (2010). Effect of the C3a-receptor antagonist SB 290157 on anti-OVA polyclonal antibody-induced arthritis. J. Pharmacol. Sci..

[12] Kildsgaard J (2000). Cutting edge: targeted disruption of the C3a receptor gene demonstrates a novel protective anti-inflammatory role for C3a in endotoxin-shock. J. Immunol..

[13] Jacob A, Bao L, Brorson J, Quigg RJ, Alexander JJ (2010). C3aR inhibition reduces neurodegeneration in experimental lupus. Lupus.

[14] Mamane Y (2009). The C3a anaphylatoxin receptor is a key mediator of insulin resistance and functions by modulating adipose tissue macrophage infiltration and activation. Diabetes.

[15] Proctor LM (2004). Comparative anti-inflammatory activities of antagonists to C3a and C5a receptors in a rat model of intestinal ischaemia/reperfusion injury. Br. J. Pharmacol..

[16] Lim J (2013). C5aR and C3aR antagonists each inhibit diet-induced obesity, metabolic dysfunction, and adipocyte and macrophage signaling. FASEB J..

[17] Pasupuleti M (2007). Preservation of antimicrobial properties of complement peptide C3a, from invertebrates to humans. J. Biol. Chem..

[18] Ames RS (2001). Identification of a selective nonpeptide antagonist of the anaphylatoxin C3a receptor that demonstrates antiinflammatory activity in animal models. J. Immunol..

[19] Reid RC (2014). Potent heterocyclic ligands for human complement C3a receptor. J. Med. Chem..

[20] Reid RC, Yau MK, Singh R, Lim J, Fairlie DP (2014). Stereoelectronic effects dictate molecular conformation and biological function of heterocyclic amides. J. Am. Chem. Soc..

[21] Kubota Y (1992). The effect of human anaphylatoxins and neutrophils on histamine release from isolated human skin mast cells. J. Dermatol..

[22] Beno BR, Yeung KS, Bartberger MD, Pennington LD, Meanwell NA (2015). A survey of the role of noncovalent sulfur interactions in drug design. J. Med. Chem..

[23] Crass T (1996). Expression cloning of the human C3a anaphylatoxin receptor (C3aR) from differentiated U-937 cells. Eur. J. Immunol..

[24] Atwood NK, Lopez J, Wager-Miller J, Mackie K, Straiker A (2011). Expression of G protein-coupled receptors and related proteins in HEK293, AtT20, BV2, and N18 cell lines as revealed by microarray analysis. BMC Genomics..

[25] Insel PA (2015). G Protein–Coupled Receptor (GPCR) expression in native cells: “novel” endoGPCRs as physiologic regulators and therapeutic targets. Mol. Pharmacol..

[26] Bokisch VA, Muller-Eberhard HJ (1970). Anaphylatoxin inactivator of human plasma: its isolation and characterization as a carboxypeptidase. J. Clin. Invest..

[27] Morris CJ (2003). Carrageenan-induced paw edema in the rat and mouse. Methods Mol. Biol..

[28] Rodewald HR, Feyerabend TB (2012). Widespread immunological functions of mast cells: fact or fiction?. Immunity.

[29] Voehringer D (2013). Protective and pathological roles of mast cells and basophils. Nat. Rev. Immunol..

[30] Wernersson S, Pejler G (2014). Mast cell secretory granules: armed for battle. Nat. Rev. Immunol..

[31] Theoharides TC, Valent P, Akin C (2015). Mast cells, mastocytosis, and related disorders. N. Engl. J. Med..

[32] Theoharides TC, Stewart JM (2015). Genitourinary mast cells and survival. Transl Androl Urol.

[33] Bischoff SC (2007). Role of mast cells in allergic and non-allergic immune responses: comparison of human and murine data. Nat. Rev. Immunol..

[34] Sarrazin S, Sieweke MH (2016). Eosinophils and mast cells: a lineage apart. Nat. Immunol..

[35] Dwyer DF, Barrett NA, Austen KF, Immunological Genome Project, C (2016). Expression profiling of constitutive mast cells reveals a unique identity within the immune system. Nat. Immunol..

[36] Minton K (2014). Innate immunity: the inside story on complement activation. Nat. Rev. Immunol..

[37] Liszewski MK (2013). Intracellular complement activation sustains T cell homeostasis and mediates effector differentiation. Immunity.

[38] March DR (2004). Potent cyclic antagonists of the complement C5a receptor on human polymorphonuclear leukocytes. Relationships between structures and activity. Mol. Pharmacol..

[39] Reid RC, Abbenante G, Taylor SM, Fairlie DP (2003). A convergent solution-phase synthesis of the macrocycle Ac-Phe-[Orn-Pro-D-Cha-Trp-Arg], a potent new antiinflammatory drug. J. Org. Chem..

[40] Ariffin JK (2016). Histone deacetylase inhibitors promote mitochondrial reactive oxygen species production and bacterial clearance by human macrophages. Antimicrob. Agents Chemother..

[41] Seow V (2013). Inflammatory responses induced by lipopolysaccharide are amplified in primary human monocytes but suppressed in macrophages by complement protein C5a. J. Immunol..

[42] Lim J (2013). Diet-induced obesity, adipose inflammation, and metabolic dysfunction correlating with PAR2 expression are attenuated by PAR2 antagonism. FASEB J..

[43] Kilkenny C, Browne W, Cuthill IC, Emerson M, Altman DG (2010). NC3Rs reporting guidelines working group. animal research: reporting in vivo experiments: the ARRIVE guidelines. Br. J. Pharmacol..

[44] Lohman RJ (2016). Differential anti-inflammatory activity of HDAC inhibitors in human macrophages and rat arthritis. J. Pharmacol. Exp. Ther..

