# Supplementary material for: Exploiting a novel conformational switch to control innate immunity mediated by complement protein C3a
Source: Nat Commun. 2017 Aug 24;8:351. doi: 10.1038/s41467-017-00414-w (PMC5570900; doi:10.1038/s41467-017-00414-w)
Supplement: Supplementary file 1 — Supplementary Information [file 41467_2017_414_MOESM1_ESM.pdf]

File name: Supplementary Information
Description: Supplementary figures, supplementary table, supplementary methods and supplementary references.

Supplementary Methods

Chemical Synthesis and Characterization

Synthesis of compounds 1 (JR4) and 2 (BR86).

Supplementary Figure 1 outlines the synthetic scheme used to prepare compounds **1** and **2** via **7** and **8,** then **9** and **10**.

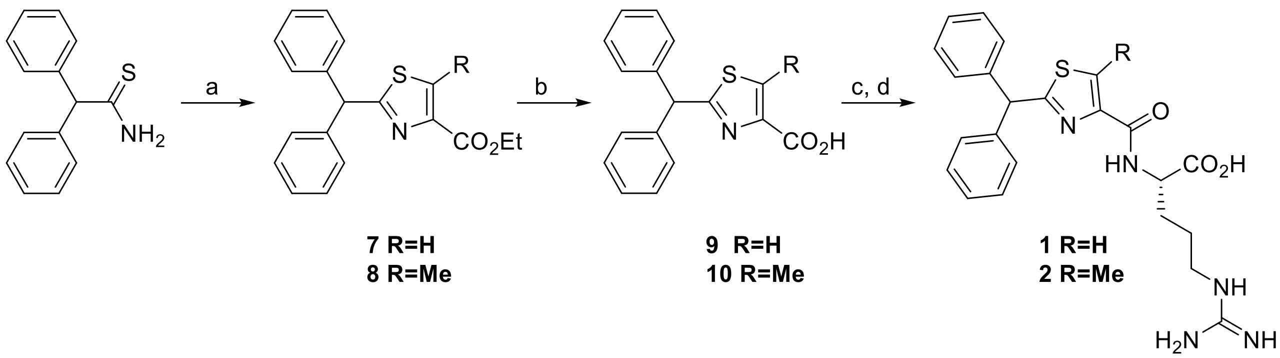

7 R=H **8** R=Me; **9** R=H **10** R=Me; **1** R=H **2** R=Me

Supplementary Figure 1. Synthesis of compounds **1** and **2**.

Reagents and conditions: a) Ethyl bromopyruvate (for **7** or ethyl 3-bromo-2-oxobutanoate for **8**), EtOAc, 80 ^{o}C, 2 h; b) NaOH, MeOH, H_2O; c) H-Arg-OEt, BOP, DIPEA, DMF; d) NaOH, MeOH, H_2O.

Ethyl 2-benzhydrylthiazole-4-carboxylate (7)

Ethyl bromopyruvate (172 mg, 110 µL, 0.88 mmol) was added to a solution of 2,2-diphenylthioacetamide[1] (200 mg, 0.88 mmol) in ethyl acetate (2 mL). The solution was refluxed for 3 hours at 80 ^{o}C, diluted with ethyl acetate (30 mL), and washed with saturated sodium bicarbonate solution (10 mL) and brine (10 mL). The organic layer was dried over magnesium sulfate, filtered and concentrated *in vacuo*. The product was purified via flash chromatography using EtOAc in Petroleum Spirit (10% - 25%) to yield the thiazole **7** (210 mg, 0.65 mmol, 74%) as a crystalline solid. ^{1}H NMR (400 MHz, $CDCl_3$): δ 8.11 (s, 1H), 7.34-7.16 (m, 10H), 5.98 (s, 1H), 4.41 (q, J = 7.2 Hz, 2H), 1.38 (t, J = 7.1 Hz, 3H) ^{13}C NMR (100 MHz, $CDCl_3$): δ 174.7, 161.5, 147.1, 141.4, 128.9, 128.6, 128.0, 127.3, 61.4, 55.0, 14.3.

2-Benzhydrylthiazole-4-carboxylic acid (9)

Sodium hydroxide (108 mg, 2.71 mmol) was added to a solution of ethyl 2-benzhydrylthiazole-4-carboxylate **7** (185 mg, 0.57 mmol) in methanol (9 mL) and water (2 mL). After one hour at room temperature, 2M hydrochloric acid (1.71 mL, 3.42 mmol) was added to the reaction and the solution extracted into diethyl ether (30 mL). The organic layer

was washed with brine (2 x 10 mL), dried over magnesium sulfate and filtered. The solution was concentrated *in vacuo* to yield the acid **9** (163 mg, 0.55 mmol, 97 %). ^{1}H NMR (400 MHz, $CDCl_3$): δ 8.21 (s, 1H), 7.36-7.20 (m, 10H), 5.92 (s, 1H) ^{13}C NMR (100 MHz, $CDCl_3$): δ 175.1, 163.6, 146.2, 141.1, 128.9, 128.9, 128.8, 127.5, 54.9.

2-Benzhydryl-5-methylthiazole-4-carboxylic acid (10)

^{1}H NMR (600 MHz, $CDCl_3$): δ 7.37-7.18 (m, 10H), 5.74 (s, 1H), 2.75 (s, 3H). ^{13}C NMR (150 MHz, $CDCl_3$): δ 169.6, 162.0, 145.8, 141.1, 140.0, 128.8, 127.5, 54.8, 12.8.

Compound 1 (JR4)

2-Benzhydrylthiazole-4-carboxylic acid **9** (49.9 mg, 0.169 mmol) was dissolved in DMF (1 mL) and H-Arg-OEt (47.1 mg, 0.171 mmol) and DIPEA (60 μL, 0.338 mmol) added with good stirring. BOP (75 mg, 0.171 mmol) was added to the homogeneous solution and the reaction was stirred at room temperature overnight. The product was confirmed by mass spectroscopy (m/z 479, MH^+) and the reaction evaporated under high vacuum for 3 h to produce the ethyl ester as a thick gum. The product was dissolved in methanol (1.3 mL) and water (1 mL) before the addition of sodium hydroxide (95 mg, 2.37 mmol). After stirring for 10 minutes at RT, the reaction had gone to completion, as observed by mass spectroscopy (m/z 452.2, MH^+), and 2M HCl (1.2 mL, 2.37 mmol) was added. TFA (3 drops) was added to the solution and the mixture purified by HPLC and lyophilised to produce the product **1** (62 mg, 0.109 mmol, 65%) as a white powder. ^{1}H NMR (600 MHz, DMSO-d_6): δ 8.28 (d, J = 8.0 Hz, 1H), 8.23 (s, 1H), 7.50 (t, J = 5.3 Hz, 1H), 7.37-7.27 (m, 10H), 6.03 (s, 1H), 4.42-4.39 (m, 1H), 3.11-3.07 (m, 2H), 1.90-1.83 (m, 1H), 1.81-1.74 (m, 1H), 1.51-1.45 (m, 2H), ^{13}C NMR (150 MHz, DMSO-d_6): δ 173.5, 173.0, 160.4, 156.6, 149.5, 141.8, 128.7, 128.67, 128.66, 127.3, 127.2, 124.9, 53.6, 51.6, 40.3, 28.0, 25.3. HRMS calculated for $C_{23}H_{26}N_5O_3S^+$ 452.1751, found 452.1750. HPLC t_R 14.6 min.

Compound 2 (BR86)

This compound is the 4-methyl analogue of compound **1** and was prepared by the same methods except that ethyl 3-bromo-2-oxobutanoate was used in place of ethyl bromopyruvate in step 1. ^{1}H NMR (600 MHz, DMSO-d_6): δ 8.14 (d, J = 8.0 Hz, 1H), 7.54 (t, J = 5.7 Hz, 1H), 7.39-7.33 (m, 4H), 7.33-7.25 (m, 6H), 5.94 (s, 1H), 4.39 (m, 1H), 3.14-3.06 (m, 2H), 2.68 (s, 3H), 1.86 (m, 1H), 1.76 (m, 1H), 1.53-1.43 (m, 2H). ^{13}C NMR (150 MHz, DMSO-d_6): δ

173.1, 168.4, 161.8, 156.6, 142.3, 141.8, 140.8, 128.7, 128.6, 127.2, 53.5, 51.3, 40.3, 28.2, 25.3, 12.2. HRMS calculated for $C_{24}H_{28}N_5O_3S^+$ 466.1907, found 466.1907. HPLC t_R 15.4 min.

Synthesis of Compound 3 (BR103)

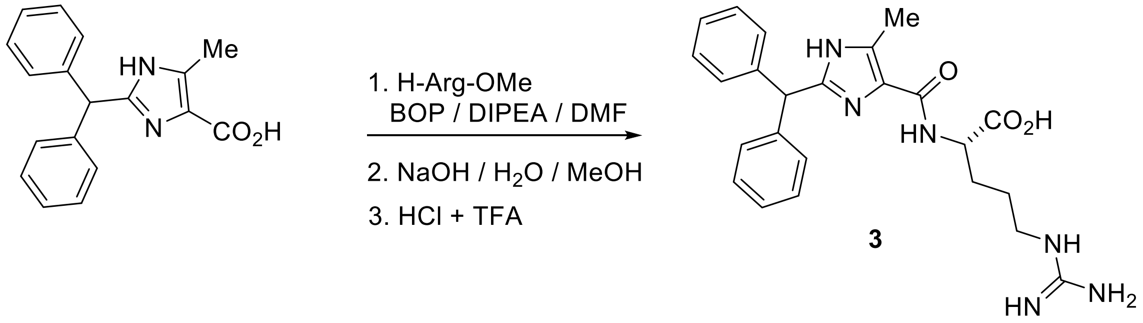

Supplementary Figure 2. Synthesis of compound **3**.

Compound **3** was prepared by an improved procedure based on our earlier methods.[1] A mixture of arginine methyl ester dihydrochloride (1.20 g, 4.60 mmol), 2-benzhydryl-5-methyl-1H-imidazole-4-carboxylic acid (880 mg, 3.00 mmol) and BOP (1.48 g, 3.35 mmol) was stirred in DMF (10 mL) for 10 min before the addition of DIPEA (1.5 mL, 8.40 mmol). Stirring was continued at room temperature for 19 h then the mixture was concentrated *in vacuo*. The residue was dissolved in MeOH (25 mL) then 2M NaOH (10 mL) was added and stirred for 1h. The mixture was acidified with 2M HCl (10 mL) and TFA (400 μL), filtered below 0.45 μm and purified by preparative reverse phase HPLC to give a white powder after lyophilisation (1.36 g, 81%, TFA salt). ^{1}H NMR (600 MHz, DMSO-d$_6$): δ 7.91 (d, J = 7.5 Hz, 1H), 7.65 (t, J = 5.6 Hz, 1H), 7.37-7.32 (m, 4H), 7.30-7.24 (m, 6H), 5.69 (s, 1H), 4.38 (m, 1H), 3.16-3.05 (m, 2H), 2.41 (s, 3H), 1.83 (m, 1H), 1.72 (m, 1H), 1.55-1.46 (m, 2H). ^{13}C NMR (150 MHz, DMSO-d$_6$): δ 173.4, 161.7, 156.8, 146.7, 140.3, 132.4, 128.7, 128.6, 127.1, 51.1, 49.4, 40.3, 28.5, 25.3, 10.6. HRMS calculated for $C_{24}H_{29}N_6O_3^+$ 449.2296, found 449.2295. HPLC t_R 11.5 min.

Synthesis of compound 4 (AY11).

Supplementary Figure 3 outlines the synthetic scheme used to prepare compound **4** via **11** and **12**.

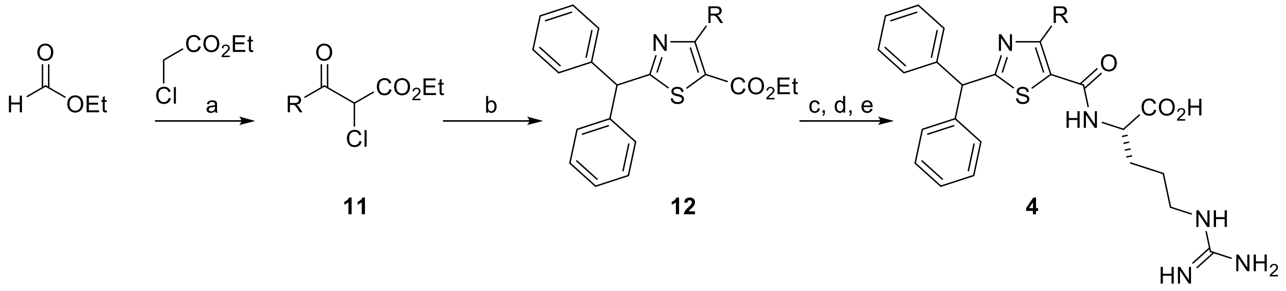

Supplementary Figure 3. Synthesis of compound **4**

Reagents and conditions: a) KO^tBu, THF, 0 °C→RT; b) 2,2-Diphenylthioacetamide, DIPEA, EtOH, microwave 100 °C, 60 min; c) NaOH, H_2O, EtOH; d) H-Arg-OEt, BOP, DIPEA, DMF; e) NaOH, H_2O, EtOH, 5 min.

Ethyl 2-chloro-3-oxopropanoate (11).

Potassium *tert*-butoxide (1.13 g, 10.1 mmol) in anhydrous THF (10 mL) was stirred at 0 °C under N_2. To this cooled solution, a mixture of solution of ethyl chloroacetate (1.11 mL, 10.1 mmol) and ethyl formate (0.81 mL, 10.1 mmol) in anhydrous THF (4 mL) was added dropwise at 0 °C over 30 min. After addition, the reaction mixture was stirred at 0°C for 2 h then warmed to room temperature and stirred overnight. Water (10 mL) was added and the reaction mixture was extracted with diethyl ether (2 × 15 mL). The aqueous layer was acidified to pH 4 with 2 M HCl and then extracted with ether (3 × 25 mL). All organic phases were combined, dried over $MgSO_4$, filtered and evaporated to dryness *in vacuo*. The crude product (ESI-MS: *m/z* 151.1 $[MH]^+$) was used immediately for the next reaction without purification.

Ethyl 2-benzhydrylthiazole-5-carboxylate (12)

Ethyl 2-chloro-3-oxopropanoate **11** (135 mg, 0.89 mmol) and 2,2-diphenylthioacetamide[1] (102 mg, 0.45 mmol) were dissolved in EtOH (4 mL) then DIPEA (78 µL, 0.45 mmol) was added. The reaction mixture was heated in a microwave at 100 °C for 1h then evaporated to dryness *in vacuo*. The residue was dissolved in MeCN/H_2O and purified by preparative HPLC to yield the thiazole **12** (20 mg, 14%). HRMS: $[MH]^+$ calc. for $C_{19}H_{18}N_1O_2S^+$ 324.1053 found 324.1053. ^1H NMR (600 MHz, $CDCl_3$): δ 8.36 (s, 1 H), 7.24–7.36 (m, 10 H), 5.82 (s, 1 H), 4.33 (q, 2 H, J = 6.9 Hz), 1.34 (t, 3 H, J = 7.1 Hz).

Compound 4 (AY11)

Ethyl 2-benzhydrylthiazole-5-carboxylate **12** (20 mg) was dissolved in EtOH/H_2O/2M NaOH (4:1:1, 0.6 mL) resulting in ester hydrolysis. The mixture was acidified and the acid was extracted into EtOAc, washed with brine, dried over $MgSO_4$ and evaporated. The crude carboxylic acid product (ESI-MS: *m/z* 296.2 $[MH]^+$) from this reaction was used for the next step without further purification. The thiazole acid (30 mg, 0.10 mmol) was coupled to H-Arg-OEt with BOP reagent (65 mg, 0.15 mmol) and DIPEA (50 μL) in DMF (1 mL) at room temperature for 12 h. DMF was removed *in vacuo* and the crude product was purified by semi-preparative HPLC (HRMS: $[MH]^+$ calc. for $C_{25}H_{30}N_5O_3S^+$ 480.2064, found 480.2065). The purified compound was treated with EtOH/H_2O/2M NaOH (4:1:1, 0.6 mL) solution to hydrolyse the ethyl ester then concentrated *in vacuo*. The residue was re-dissolved in MeCN/H_2O, acidified with TFA and purified by semi-preparative HPLC to give compound **4** (3 mg, 12%). HRMS: $[MH]^+$ calc. for $C_{23}H_{26}N_5O_3S^+$ 452.1751, found 452.1751. ^{1}H NMR (600 MHz, DMSO-d_6): δ 8.84 (d, 1 H, J = 7.8 Hz), 8.43 (s, 1 H), 7.61 (t, 1 H, J = 5.5 Hz), 7.29–7.37 (m, 10 H), 6.00 (s, 1 H), 4.32–4.35 (m, 1 H), 3.08–3.15 (m, 2 H), 1.82–1.88 (m, 1 H), 1.67–1.73 (m, 1 H), 1.49–1.59 (m, 2 H). ^{13}C NMR (150 MHz, DMSO-d_6): δ 176.9, 173.2, 160.0, 156.6, 143.9, 141.7, 134.6, 128.71, 128.67, 127.20, 53.8, 52.0, 40.3, 27.7, 25.4. HPLC t_R 14.2 min.

Synthesis of Compound 5 (BR91)

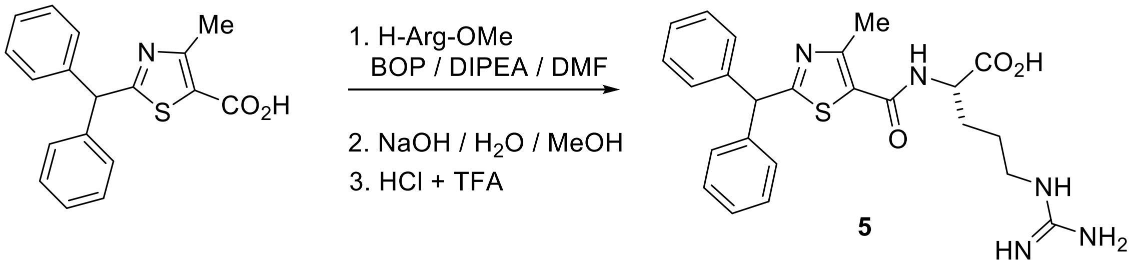

Supplementary Figure 4. Synthesis of compound **5**.

Compound **5** was prepared from 2-benzhydryl-4-methylthiazole-5-carboxylic acid using the same procedure as compound **3** giving a white powder (TFA salt) after lyophilisation in 73% yield. ^{1}H NMR (600 MHz, DMSO-d_6): δ 8.41 (d, J = 7.8 Hz, 1H), 7.52 (t, J = 5.6 Hz, 1H), 7.39-7.26 (m, 10H), 5.94 (s, 1H), 4.29 (m, 1H), 3.13-3.04 (m, 2H), 2.52 (s, 3H), 1.81 (m, 1H), 1.67 (m, 1H), 1.57-1.44 (m, 2H). ^{13}C NMR (150 MHz, DMSO-d_6): δ 173.1, 172.6,

161.4, 156.6, 154.9, 141.7, 128.7, 127.2, 125.5, 53.7, 52.2, 40.3, 27.5, 25.4, 17.0. HRMS calculated for $C_{24}H_{28}N_5O_3S^+$ 466.1907, found 466.1907. HPLC t_R 14.3 min.

Synthesis of compound 6 (BR111)

Supplementary Figure 5 outlines the synthetic scheme used to prepare compound **6** from **13** via **14**.

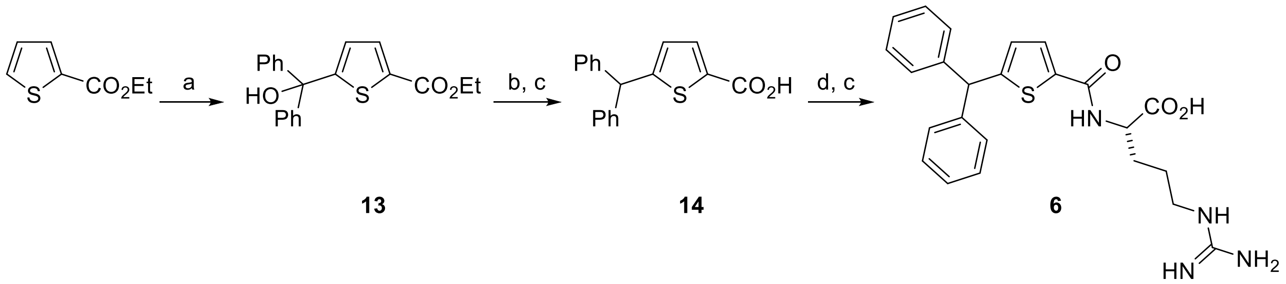

Supplementary Figure 5. Synthesis of compound **6** (BR111)

Reagents and conditions: a) LDA, THF, -78 °C, benzophenone. b) TFA, Et_3SiH. c) NaOH, H_2O, MeOH. d) BOP, DIPEA, H-Arg-OMe, DMF.

Ethyl 5-(hydroxydiphenylmethyl)thiophene-2-carboxylate (13)

A solution of ethyl thiophene-2-carboxylate (730 mg, 4.68 mmol) in dry THF (10 mL) was stirred at -78 °C under N_2 then LDA (2M in THF/heptane 2.5 mL) was added followed by a solution of benzophenone (1.00 g, 5.50 mmol) in THF (5 mL). The mixture was allowed to warm to room temperature and stirring was continued for 2 h before the reaction was quenched by the addition of 2M HCl. The mixture was extracted with EtOAc and the extracts were washed with 10% $NaHCO_3$, brine and dried over $MgSO_4$. After removal of solvent the residual oil was purified by flash chromatography eluting with 10-25% EtOAc/petrol to give the ester **13** as a pale yellow oil 1.14 g, 72%. ^{1}H NMR (600 MHz, $CDCl_3$): δ 7.66 (d, J = 3.9 Hz, 1H), 7.38-7.29 (m, 10H), 6.78 (d, J = 3.9 Hz, 1H), 4.32 (q, J = 7.1 Hz, 2H), 1.34 (t, J = 7.1 Hz, 3H).

5-Benzhydrylthiophene-2-carboxylic acid (14)

Ethyl 5-(hydroxydiphenylmethyl)thiophene-2-carboxylate **13** (1.00 g, 2.96 mmol) was dissolved in a mixture of TFA (5 mL) and triethylsilane (0.5 mL). The solution was stirred at room temperature for 30 min then evaporated under a stream of N_2. The residue was dissolved in DCM and washed with 10% $NaHCO_3$ then evaporated. The residue was

dissolved in EtOH (12 mL) and a solution of NaOH (200 mg in water 3 mL) was added. The mixture was refluxed for 1h then acidified with 2M HCl and extracted with DCM. The DCM was evaporated and the residue was triturated with petrol to give a light brown powder (820 mg, 94%). ^{1}H NMR (600 MHz, $CDCl_3$): δ 7.73 (d, J = 3.9 Hz, 1H), 7.36-7.17 (m, 10H), 6.77 (dd, J = 3.9, 0.8 Hz, 1H), 5.68 (s, 1H). ^{13}C NMR (150 MHz, $CDCl_3$): δ 167.4, 157.9, 142.6, 135.0, 131.3, 128.8, 128.7, 127.5, 127.2, 52.7. ESMS $[M-H]^-$ 293.0.

Compound 6 (BR111)

A mixture of 5-benzhydrylthiophene-2-carboxylic acid **14** (124 mg, 0.421 mmol), BOP reagent (195 mg, 0.441 mmol) and H-Arg-OMe dihydrochloride (165 mg, 0.635 mmol) was dissolved in DMF (2 mL) then DIPEA (250 μL, 1.44 mmol) was added. The mixture was stirred at room temperature for 17 h then concentrated under vacuum to a viscous gum. The residue was dissolved in MeOH (5 mL) and 2M NaOH (2 mL) was added. The mixture was stirred for 1h then acidified with 2M HCl (2 mL) and TFA (100 μL), filtered and purified by reverse phase HPLC to give **6** as a white powder (133 mg, 56%, TFA salt) after lyophilisation. ^{1}H NMR (600 MHz, DMSO-d_6): δ 8.60 (d, J = 8.0 Hz, 1H), 7.73 (d, J = 3.8 Hz, 1H), 7.58 (t, J = 5.5 Hz, 1H), 7.37-7.22 (m, 11H), 6.79 (dd, J = 3.8, 1.0 Hz, 1H), 5.83 (s, 1H), 4.32 (m, 1H), 3.16-3.04 (m, 2H), 1.85 (m, 1H), 1.70 (m, 1H), 1.61-1.46 (m, 2H), ^{13}C NMR (150 MHz, DMSO-d_6): δ 173.4, 161.2, 156.7, 153.0, 143.2, 137.8, 128.6, 128.5, 126.9, 51.9, 51.2, 40.3, 27.7, 25.4. HRMS calculated for $C_{24}H_{27}N_4O_3S^+$ 451.1798, found 451.1798. HPLC t_R 15.2 min.

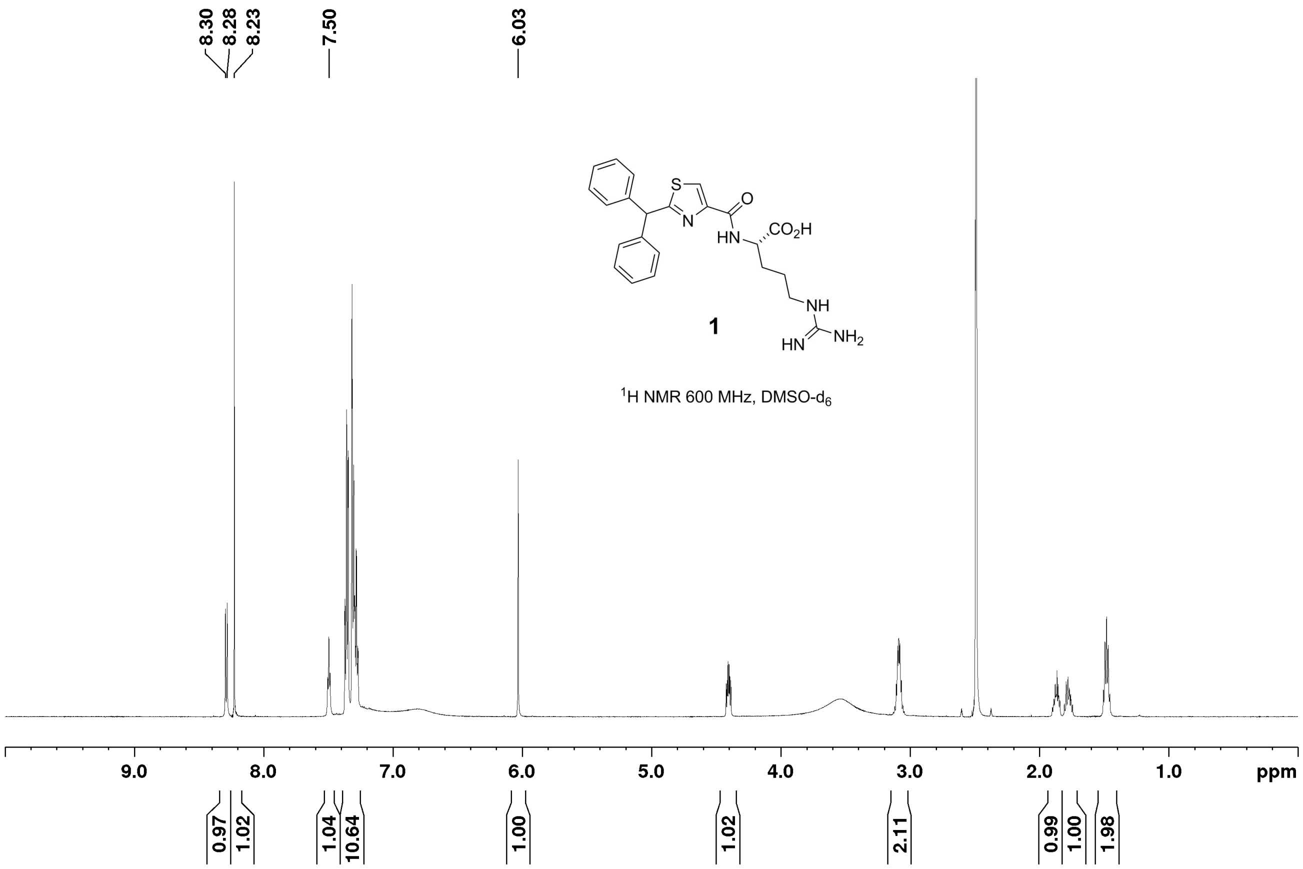

Supplementary Figure 6. Proton NMR spectrum of compound **1** in DMSO-d_6.

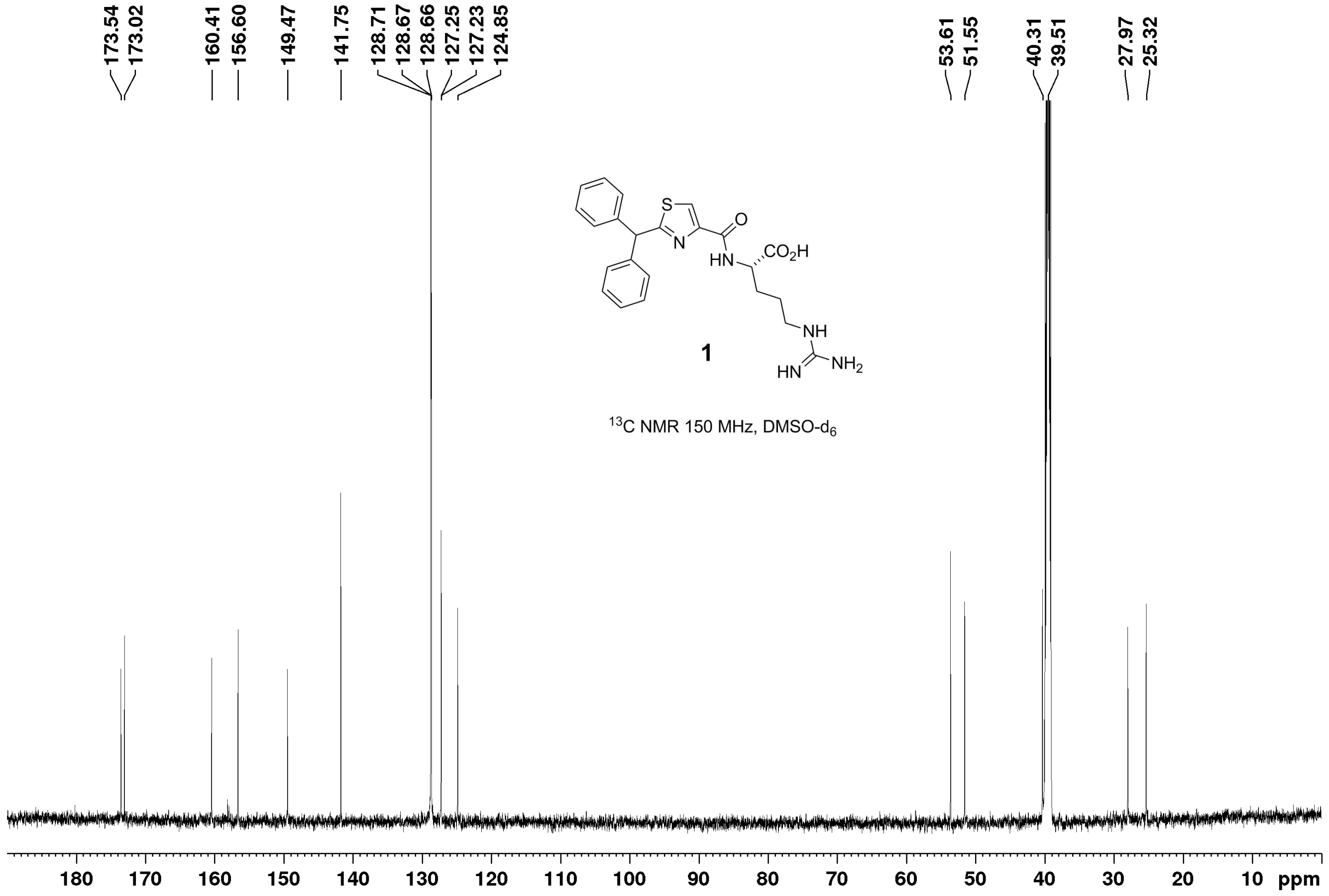

Supplementary Figure 7. Carbon NMR spectrum of compound **1** in DMSO-d_6.

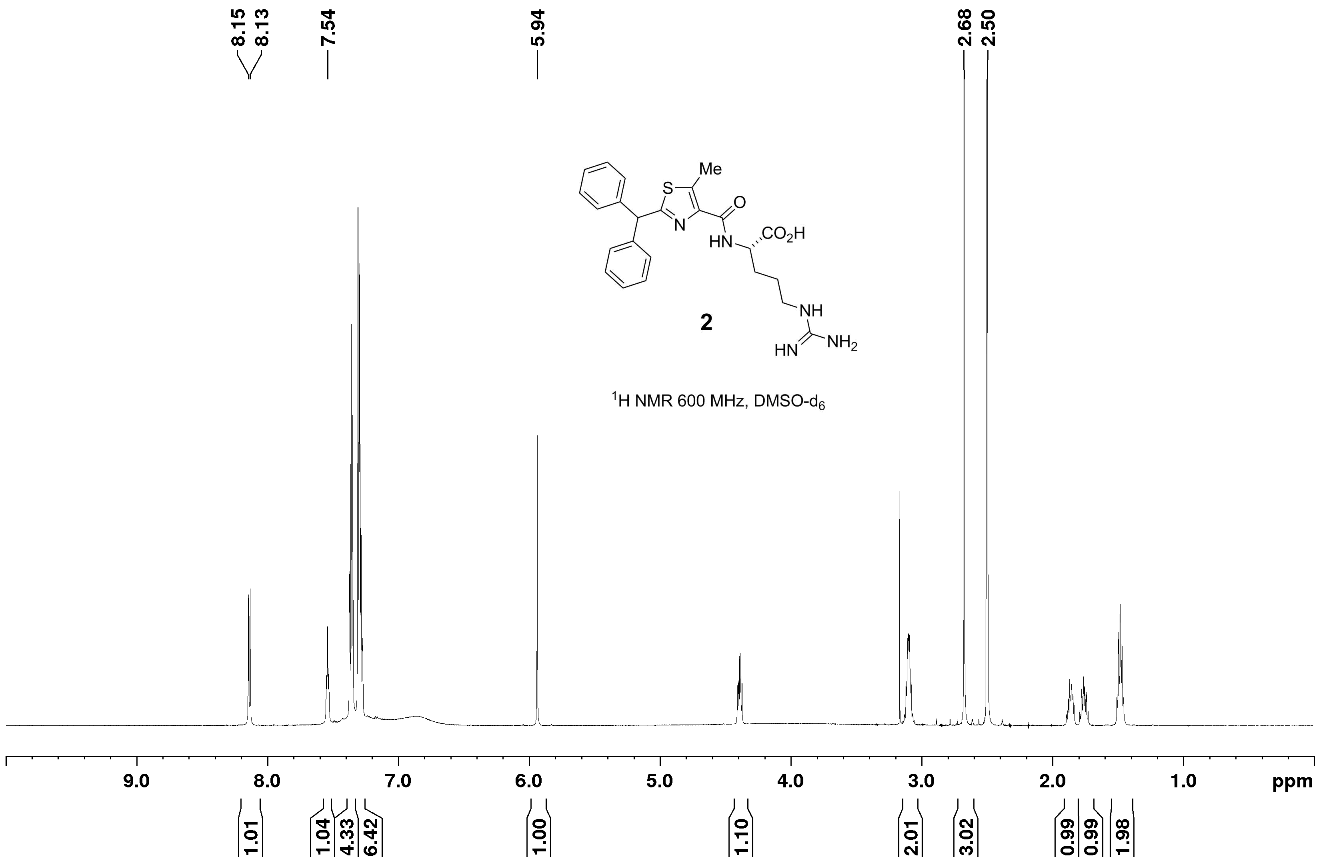

Supplementary Figure 8. Proton NMR spectrum of compound **2** in DMSO-d_6.

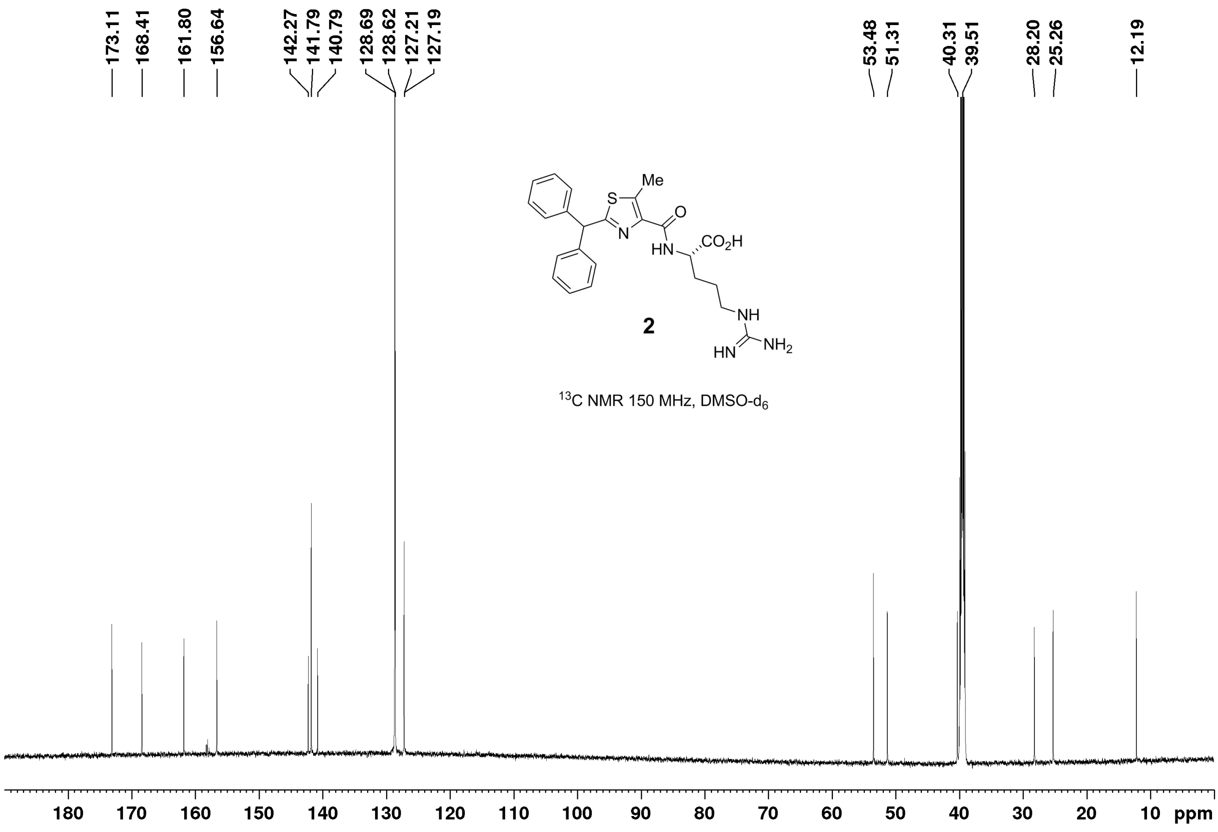

Supplementary Figure 9. Carbon NMR spectrum of compound **2** in DMSO-d_6.

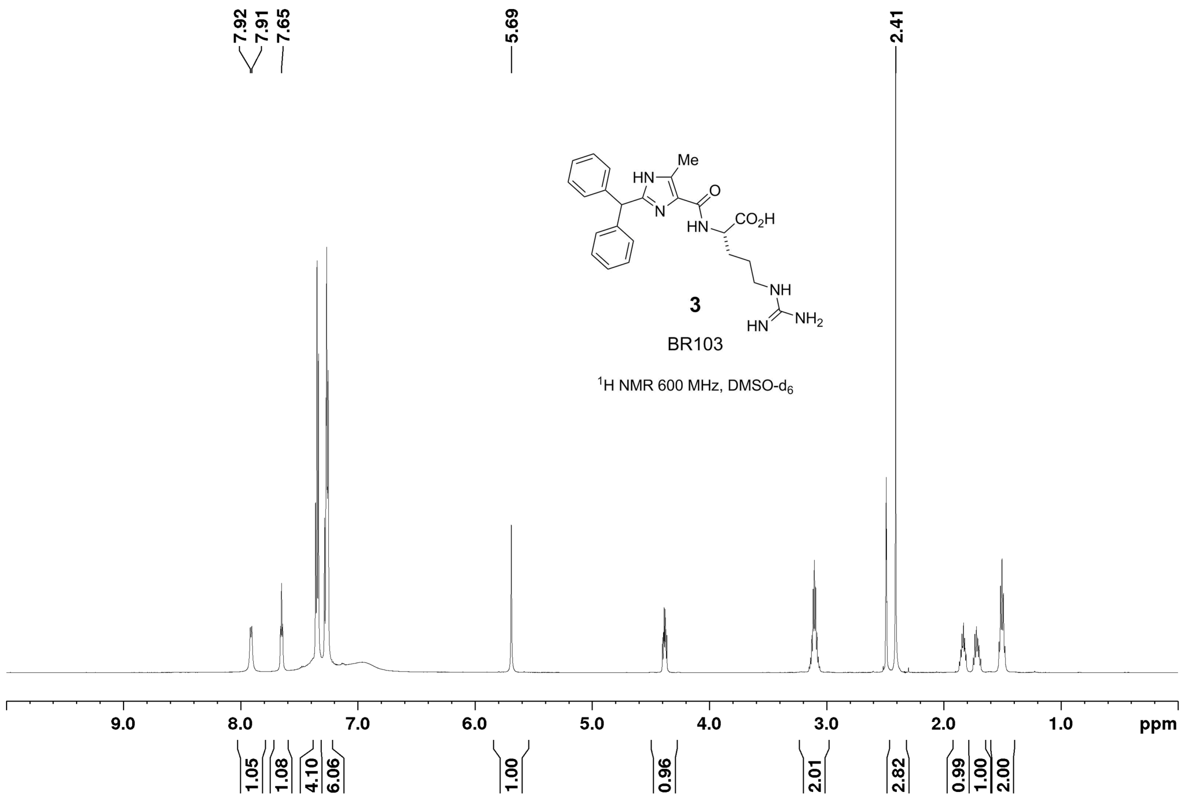

Supplementary Figure 10. Proton NMR spectrum of compound **3** in DMSO-d_6.

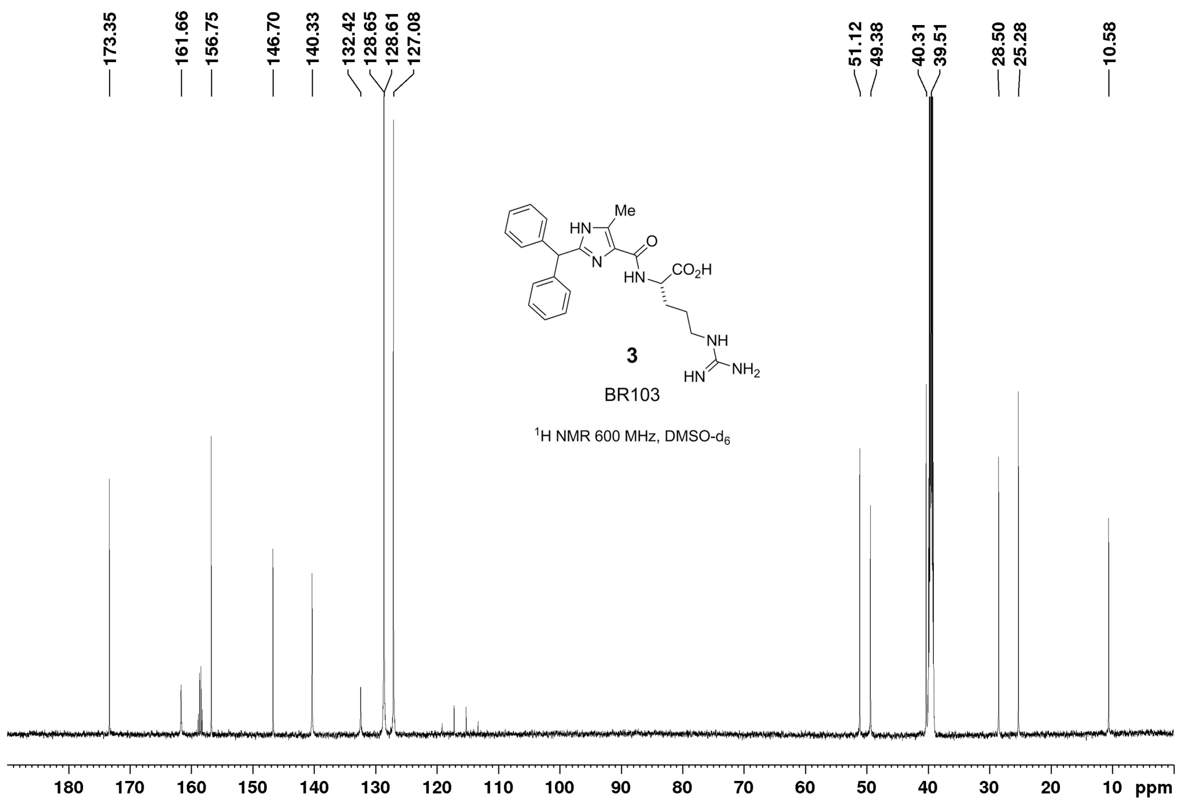

Supplementary Figure 11. Carbon NMR spectrum of compound **3** in DMSO-d_6.

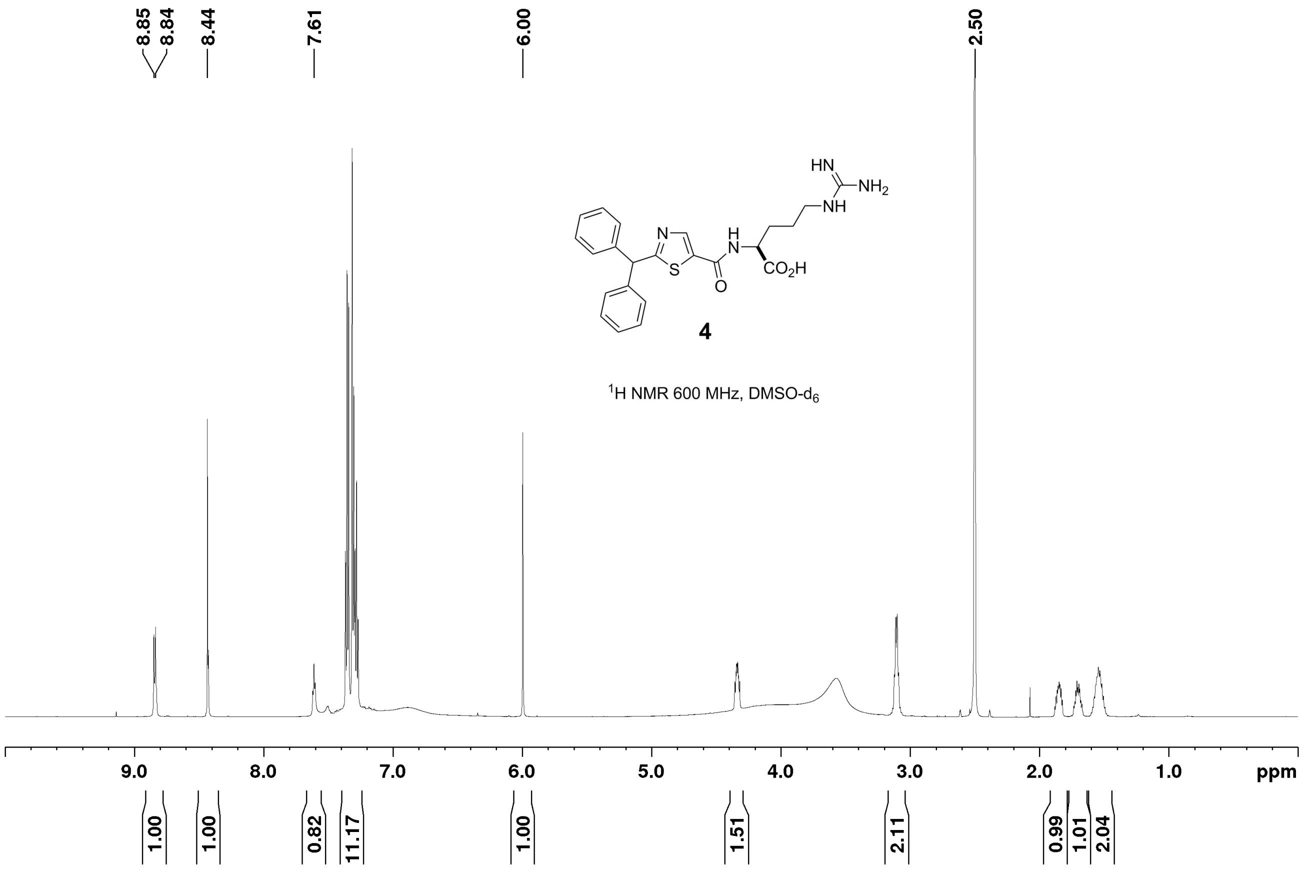

Supplementary Figure 12. Proton NMR spectrum of compound **4** in DMSO-d_6.

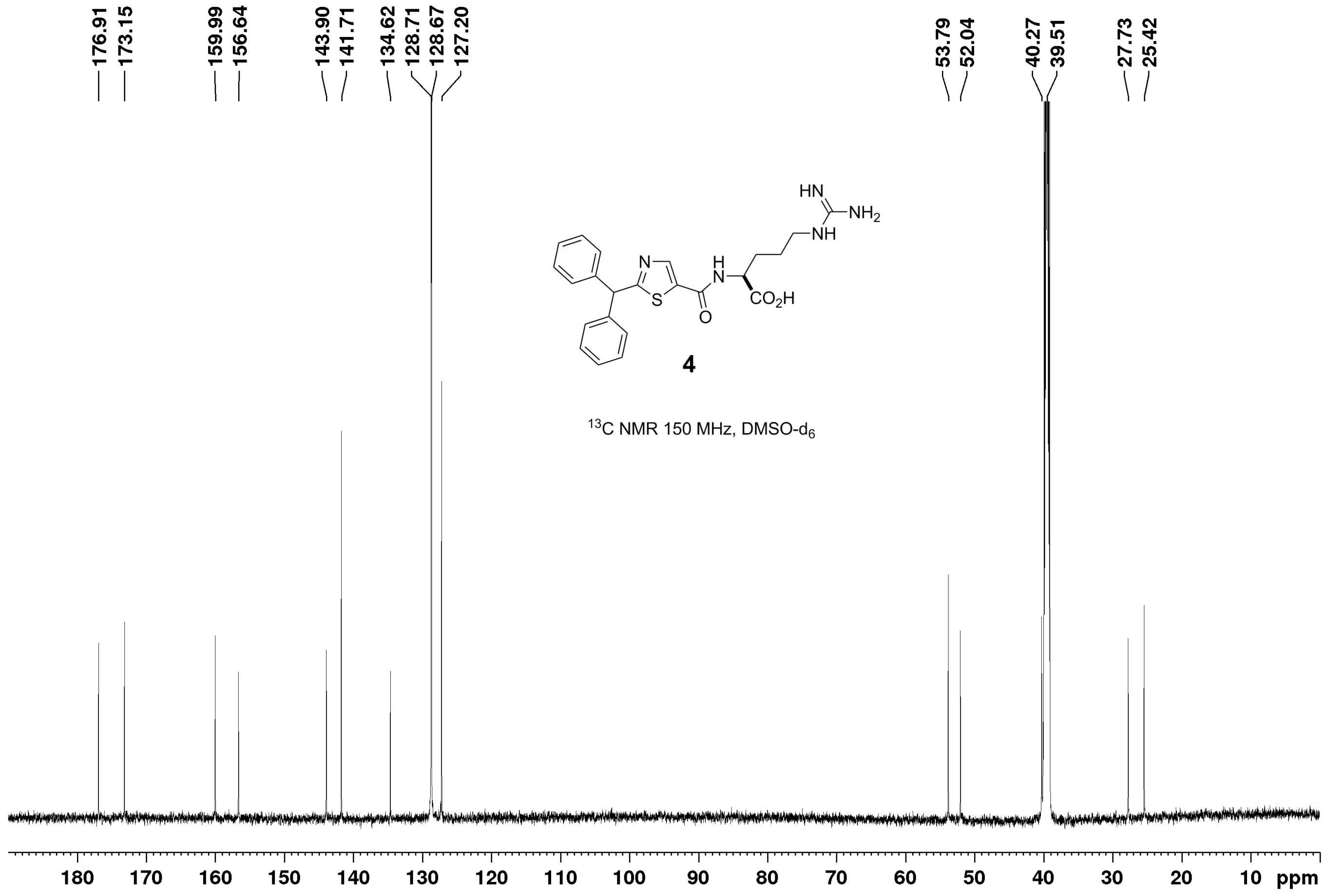

Supplementary Figure 13. Carbon NMR spectrum of compound **4** in DMSO-d_6.

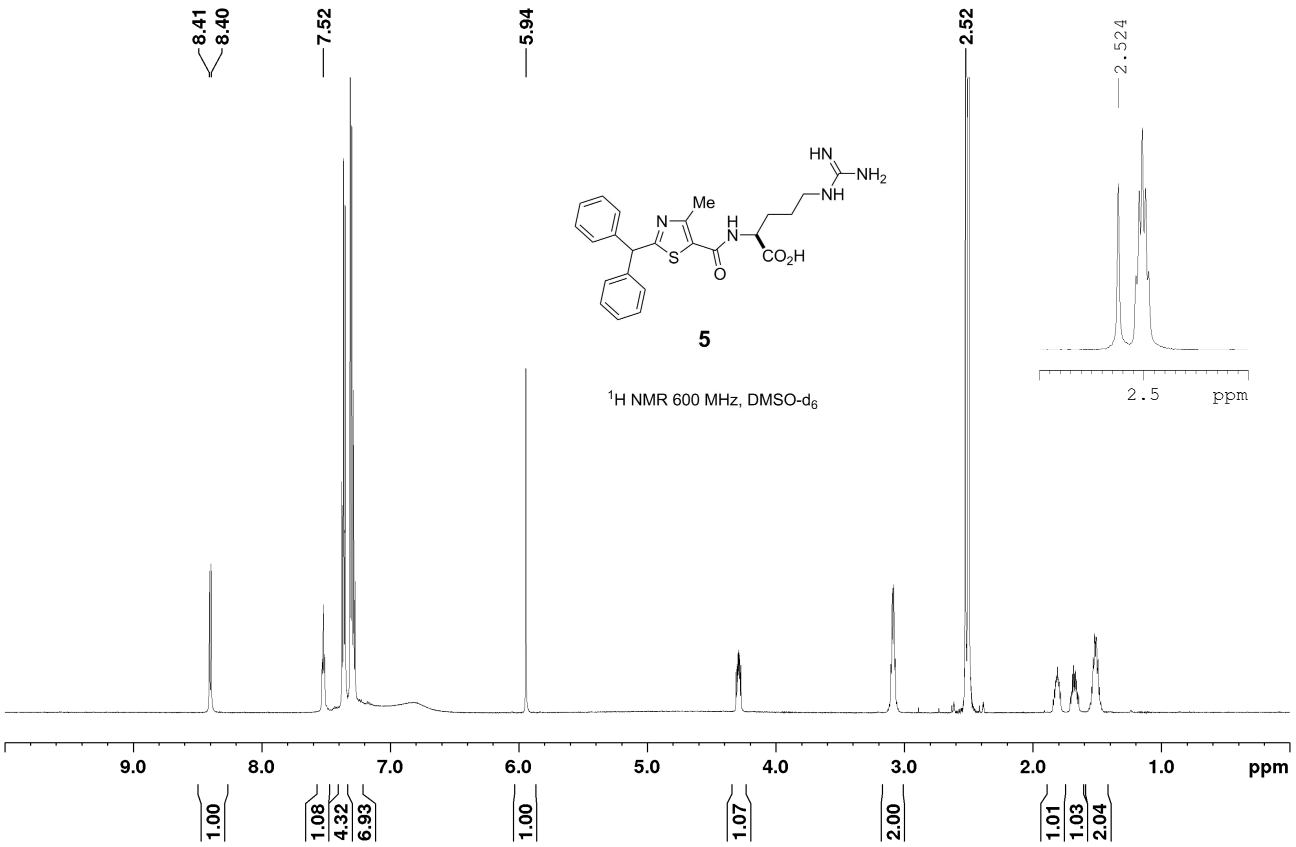

Supplementary Figure 14. Proton NMR spectrum of compound **5** in DMSO-d_6.

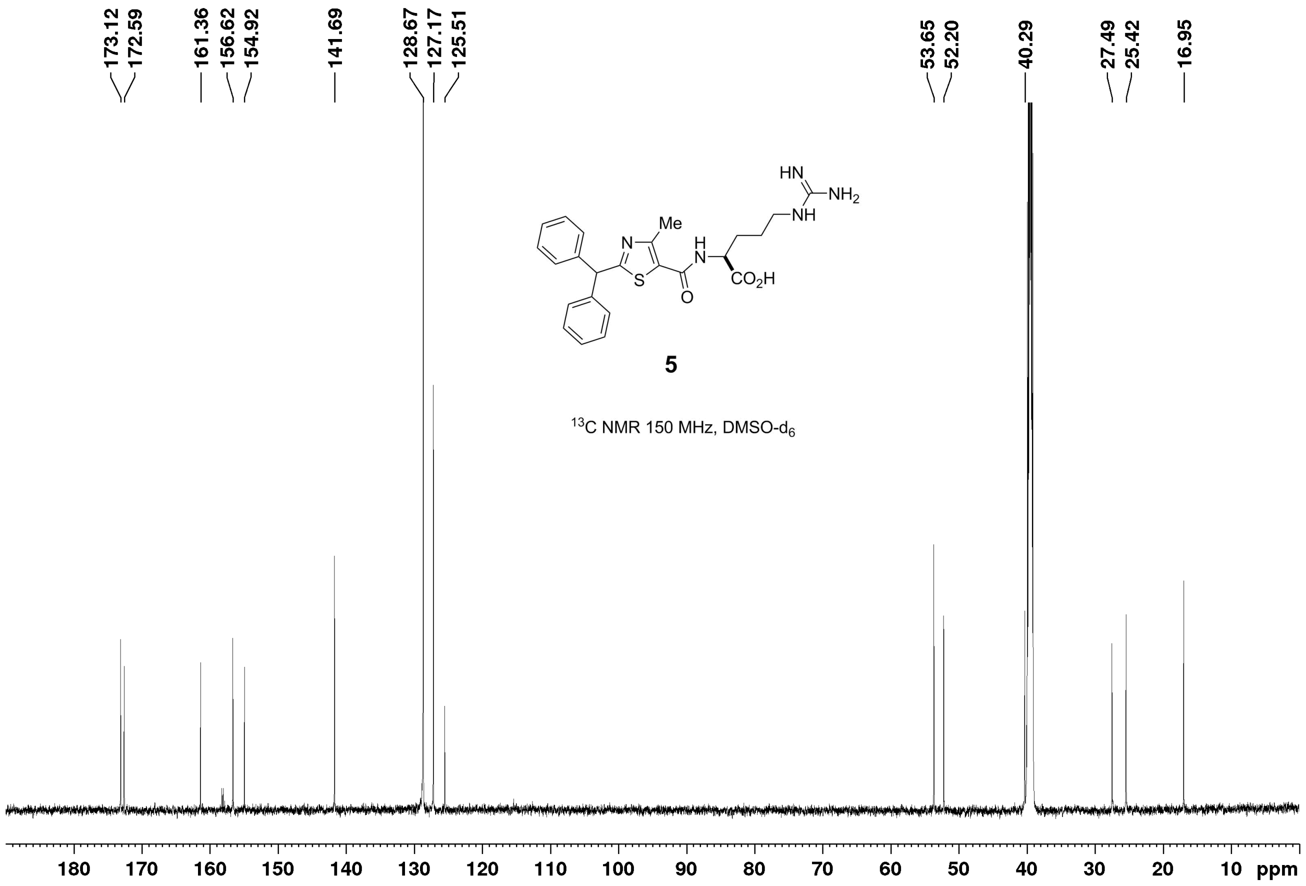

Supplementary Figure 15. Carbon NMR spectrum of compound **5** in DMSO-d_6.

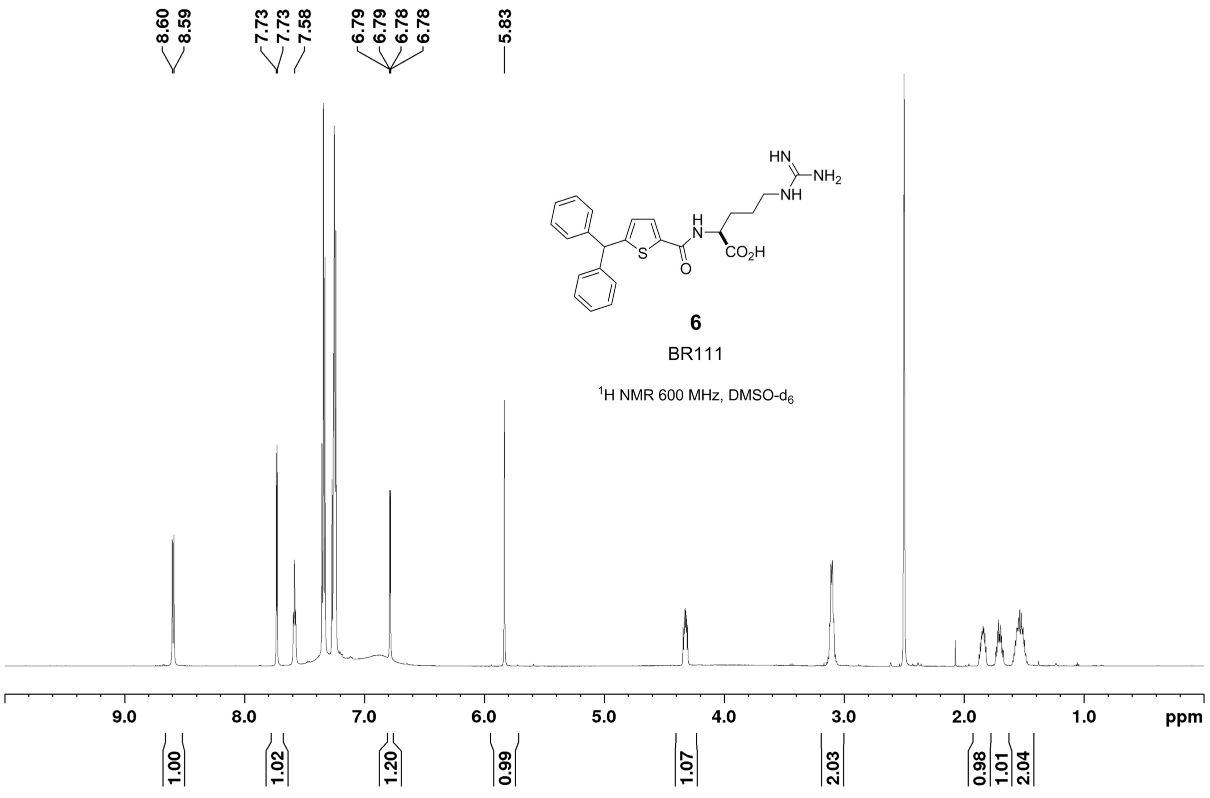

Supplementary Figure 16. Proton NMR spectrum of compound **6** in DMSO-d_6.

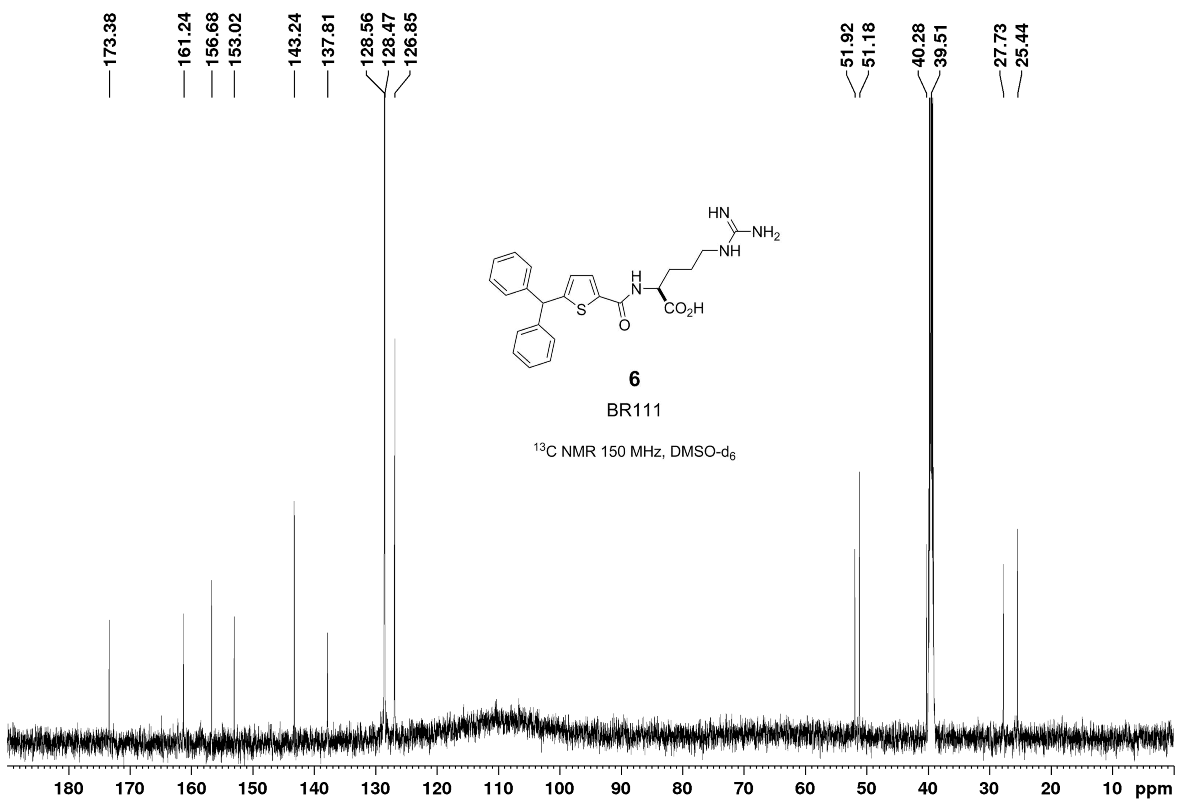

Supplementary Figure 17. Carbon NMR spectrum of compound **6** in DMSO-d_6.

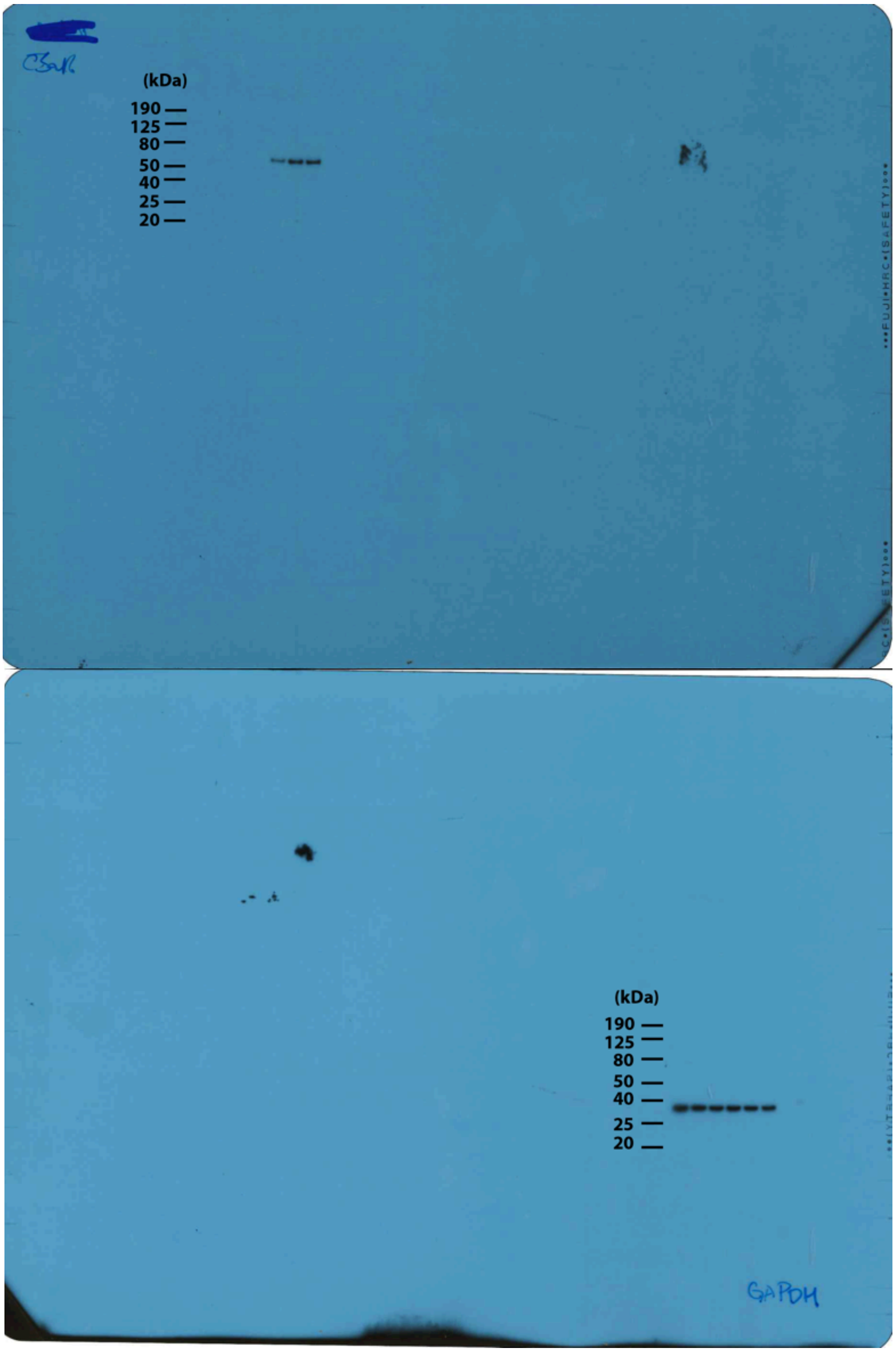

Supplementary Figure 18. Full blots of human C3aR (top) and GAPDH (bottom) of HEK293 cells transfected with human C3aR. Human C3aR was detected with mouse monoclonal IgG_{2a} (Santa Cruz Biotechnology, SC-133172) and secondary HRP-linked anti-mouse IgG (Cell Signaling Technology, #7076). GAPDH were detected using affinity isolated rabbit anti-GAPDH (Sigma-Aldrich, G9545) and secondary HRP-linked anti-rabbit IgG (Cell Signaling Technology, 7074).

Supplementary Table 1. Primer sequences for targeted rat inflammatory genes.

Name	Forward	Reverse
**Rat primers**		
Il1b	GCAGCTTTCGACAGTGAGGAGAA	GCCCAAGTCAAGGGCTTGGAA
Il6	TCTGTCTCGAGCCCACCAGGAA	GGCAACTGGCTGGAAGTCTCTTGC
Tnf	CCGTAGCCCACGTCGTAGCAAA	ATTGGCCAGGAGGGCGTTGG
Ccl3	CACCGCTGCCCTTGCTGTTCT	CAGTCGGGGTGTCAGCTCCATATGG
Mcp1	CACGCTTCTGGGCCTGTTGT	AGTGGGGCATTAACTGCATCTGG
18s	CGGCCGGTACAGTGAAACTGC	GCGCCCGTCGGCATGTATTA

Supplementary References

Reid, R. C.; Yau, M.-K.; Singh, R.; Lim, J.; Fairlie, D. P. Stereoelectronic Effects Dictate Molecular Conformation and Biological Function of Heterocyclic Amides. *J. Am. Chem. Soc*. **2014**, *136*, 11914-11917.
